# Immune-Mediated Gut-Lung Axis in IPF: Bidirectional Mendelian Randomization and Mediation Analysis of Microbiota, Inflammatory Proteins, and Metabolites

**DOI:** 10.2174/0118715303416212251126093242

**Published:** 2026-06-03

**Authors:** Jiaxing Zhao, Zenghui Liu, Lu Kuang, Aijia Dong, Heyue Gong, Huayu Yin, Shaoguo Wu, Dabin Liu, Xuehui Liu, Shuyan Liu, Limei Wu

**Affiliations:** 1 Department of Laboratory Medicine, the Affiliated Guangzhou Twelfth People's Hospital, Guangzhou Medical University, Guangzhou, Guangdong, China;; 2 Department of Immunology, Mudanjiang Medical University, Mudanjiang, Heilongjiang, China

**Keywords:** Immune cells, gut microbiota, inflammatory proteins, idiopathic pulmonary fibrosis, plasma lipidome, plasma metabolites, mendelian randomization, mediation analysis

## Abstract

**Introduction:**

Immune dysregulation plays a critical role in idiopathic pulmonary fibrosis (IPF), but the mechanisms underlying this dysregulation remain unclear.

**Methods:**

We performed bidirectional Mendelian randomization (MR) and mediation analysis using genome-wide association studies (GWAS) summary data from FinnGen (731 immune cell phenotypes, 473 gut microbiota, 91 inflammatory proteins, 179 plasma lipidome, 1,400 metabolites, and IPF cases). Sensitivity analyses ensured robustness.

**Results:**

Nine immune cell phenotypes, nine gut microbiota taxa, three inflammatory proteins, one lipid species, and 29 metabolites showed significant causal associations with IPF. Regulatory T cells (Tregs) showed a dual role: protective subsets reduced IPF risk, while activated CD4+ Tregs increased susceptibility. Mediation analysis revealed that the effect of activated CD4+ Tregs was partially mediated by *Phylum SAR324* (16.8%), TNFSF12 (10.8%), and CCL25 (4.94%). Immune cells, such as HLA-DR+ CD33- myeloid cells, modulated IPF *via* plasma metabolites (LIFR (22.1%) and decadienedioic acid (C10:2-DC) (8.38%)). Additionally, CD39+ CD4+ T cells demonstrated protective properties by suppressing the pro-fibrotic *species CHKCI006 sp900018345* (14.8%), while CD39+ CD8br Tregs promoted the anti-fibrotic factor X-16935 (5.68%).

**Discussion:**

These findings enhance our understanding of IPF pathogenesis, suggesting that modulating key metabolic pathways and immune regulatory nodes, such as Tregs, could represent promising therapeutic approaches. However, additional experimental studies are needed to validate these potential causal relationships.

**Conclusion:**

This MR study systematically maps multi-omics interactions in IPF, identifying novel mediators and therapeutic targets. Validating these mechanisms in experimental models could advance precision immunomodulatory strategies for IPF.

## 1 INTRODUCTION

Idiopathic pulmonary fibrosis (IPF) is a chronic, progressive fibrotic lung disease characterized by aberrant fibroblast activation, excessive extracellular matrix (ECM) deposition, and destruction of alveolar architecture, ultimately leading to respiratory failure [1-3]. The pathogenesis of IPF is complex and involves genetic, environmental, and immune regulatory factors [4, 5]. Current management relies on antifibrotic agents such as pirfenidone and nintedanib, yet these therapies fail to halt disease progression or reverse established fibrosis, often resulting in continued respiratory decline and a median survival of only 3–5 years despite treatment [6-8]. These limitations primarily stem from insufficient targeting of dysregulated Transforming Growth Factor-β (TGF-β)/Smad signaling, aberrant fibroblast activation, and impaired alveolar epithelial repair mechanisms, underscoring the urgent need for innovative therapies that address the core pathogenic drivers of pulmonary fibrosis [9-11].

Immune cells are central components of the human immune system, maintaining tissue homeostasis and defending against pathogens [12]. They perform diverse functions—including pathogen recognition, cytotoxic killing, and immune regulation—to ensure balanced immune responses [12-15]. Beyond their roles in host defense, immune cells critically influence tissue repair, inflammation resolution, and metabolic regulation, highlighting their systemic importance in human health [16-18]. Emerging evidence indicates that dysregulated immune activity, particularly macrophage polarization and T cell dysfunction, may drive fibrotic progression in IPF. Myeloid cells such as monocytes and alveolar macrophages can adopt pro-fibrotic phenotypes, exacerbating tissue damage through TGF-β secretion [19]. Consequently, immune modulation has emerged as a promising therapeutic strategy for fibrotic diseases, including IPF. However, the relationship between specific immune cell subsets and IPF remains incompletely defined, as only a limited range of immune phenotypes has been thoroughly investigated.

Mendelian randomization (MR) is an analytical approach that uses genetic variants as instrumental variables (IVs) to mimic the randomization process of controlled experiments [20, 21]. By leveraging the random assortment of alleles during meiosis, MR enables causal inference between exposures and health outcomes with rigor comparable to randomized controlled trials (RCTs) [22]. As a powerful epidemiological tool, MR provides robust causal estimates while minimizing confounding and reverse causation—common limitations of observational studies—and circumvents the ethical and financial constraints of traditional RCTs [23].

In this study, we hypothesized that immune dysregulation contributes to IPF progression through gut–lung axis interactions mediated by inflammatory proteins, lipids, and metabolites. To test this hypothesis, we implemented an integrative MR framework to systematically evaluate the causal relationships between these biological factors and IPF. Mediation analyses were conducted to determine whether these intermediates mediate the effects of immune dysregulation on IPF. In parallel, reverse MR analyses were performed to examine potential reverse causation, thereby strengthening the robustness of our causal inferences.

## 2 METHODS

### 2.1 Study Design

This study employed a two-sample MR design, a genetic epidemiological approach that uses instrumental variable analysis to infer causal relationships. The analysis utilized publicly available genome-wide association study (GWAS) summary statistics from European-ancestry populations. This study adheres to the Strengthening the Reporting of Observational Studies in Epidemiology (STROBE) guidelines for Mendelian Randomization (STROBE-MR) [24]. Fig. (**1**) illustrates the flowchart of this study.

### 2.2 Data Sources

The GWAS summary statistics for IPF were sourced from the FinnGen project's extensive European population cohort, specifically utilizing the most recent R12 IPF dataset (released November 4, 2024), which comprised 2,700 IPF cases and 494,583 controls [25]. Immune cell trait data were extracted from Orrù *et al*. comprehensive GWAS analysis of 731 immune cell traits in 3,757 European participants [26]. This dataset encompassed four principal immune feature categories: absolute cell counts (n=118), relative cell counts (n=192), median fluorescence intensity reflecting surface antigen levels (MFI/SALs, n=389), and morphological parameters (n=32), spanning diverse immune cell populations such as B cells, dendritic cells, T cells, monocytes, and regulatory T cells (dataset IDs: GCST90001391–GCST90002121). GWAS data on gut microbiota were obtained from the EBI database (https://www.ebi.ac.uk/gwas/), comprising fecal sample analyses from 5,959 Finnish individuals and 473 microbial taxa across taxonomic ranks from phylum to species (IDs: GCST90032172–GCST90032644) [27]. Inflammatory protein profiles were obtained from Zhao *et al*. plasma proteome study of 14,824 Europeans, identifying 91 inflammation-associated proteins *via* pQTL analysis (IDs: GCST90274758–GCST90274848) [28]. Lipidomic data originated from Ottensmann *et al*. investigation of 7,174 Europeans, quantifying 179 lipids across four classes (glycerides, glycerophospholipids, sphingolipids, sterols; IDs: GCST90277238–GCST90277416) [29]. Finally, plasma metabolite measurements were derived from Chen *et al*. cohort (n=8,299 Europeans), where 1,091 metabolites and 309 ratios were quantified *via* UPLC-MS/MS (IDs: GCST90199621–GCST90201020) [30]. Exposure and outcome data for qualified SNPs were harmonized using the IEU OpenGWAS and FinnGen databases, during which ambiguous palindromic SNPs (A/T or G/C alleles) were removed to prevent allele misalignment [31].

All GWAS data used in this study were obtained from publicly available summary-level datasets. The original studies providing these data had received ethical approval from relevant institutional review boards, and all participants had given informed consent. Since our analysis involved only secondary use of existing datasets, additional ethical approval was not required [32]. To avoid potential bias from sample overlap, we ensured that the IPF case-control cohorts were entirely independent from the datasets used for immune cells, gut microbiota, inflammatory proteins, lipidomics, and metabolomics. This approach strengthened the reliability of our causal inferences.

### 2.3 SNP Selection and Instrumental Variable Validation

Adhering to widely accepted MR selection criteria, we selected single-nucleotide polymorphisms (SNPs) significantly associated with immune cells, inflammatory proteins, plasma lipids, metabolites, and IPF status (*P* < 5×10^−8^) as well as with gut microbiota (*P* < 5×10^−6^) to serve as IVs [33-35]. To ensure independence between IVs, we applied stringent linkage disequilibrium (LD) clumping (r^2^ < 0.001 within a 10,000 kb window) [36]. Variants with minor allele frequency < 0.01 were excluded to avoid population stratification bias [37]. IV strength was evaluated using the F-statistic, and SNPs with F < 10 were removed to prevent weak instrument bias [38]. Outcome-associated SNPs were extracted from the IEU OpenGWAS and FinnGen databases [39]. Potential pleiotropic variants (*e.g.*, how specific metabolites mediate immune cell function) were identified using LDtrait (https://ldlink.nih.gov) and subsequently removed from the analysis [40].

For each SNP, we extracted the following information: chromosomal position, effect allele (EA), other allele (OA), effect allele frequency (EAF), effect size (β), standard error (SE), and Association *P*-value [41]. The odds ratio (OR) was used to quantify the strength of the association between genetic IVs and a binary outcome. The proportion of variance explained (R^2^) was calculated as: R^2^ = 2×EAF× (1-EAF) ×β^2^ / (2×EAF×(1-EAF) ×β^2^ + 2×EAF×(1-EAF) ×N×SE^2^), where N represents the effective sample size. Instrument strength was assessed using: F = R^2^×(N-2)/(1-R^2^) [42].

### 2.4 MR Analysis

All analyses were performed in R (v4.4.2) with the MR-PRESSO (v1.0) and TwoSampleMR (v0.6.8) packages. Causal estimation methods were selected based on SNP availability: When only one SNP was available, we employed the Wald ratio method for estimation; whereas in cases where two or more SNPs were available, the inverse variance weighted (IVW) method was used as the primary approach to assess the causal relationship between exposure and outcome [43]. Complementary MR approaches were implemented for sensitivity analyses, including MR-Egger regression, simple mode, weighted median, and weighted mode [44]. The Steiger directionality test was used to assess whether the hypothesized causal direction was consistent with the data. Quality control measures included: heterogeneity assessment (Cochran's Q test, *P*<0.05 threshold), pleiotropy evaluation (MR-PRESSO global test and MR-Egger intercept, *P*>0.05 indicating no bias), and leave-one-out analysis for identification of influential SNPs [45]. We implemented multiple testing corrections using two complementary approaches. First, multiple testing correction employed Bonferroni-adjusted thresholds: setting the following adjusted *P*-value thresholds: 6.84×10^−5^ (0.05/731), 1.06×10^−4^ (0.05/473), 3.57×10^−5^ (0.05/1400), 2.79×10^−4^ (0.05/179), and 5.49×10^−4^ (0.05/91) [46]. Associations meeting *P*<0.05 but not surpassing corrected thresholds were classified as suggestive. Second, to account for potential inflation of type I error rates due to multiple testing, we adjusted the primary IVW estimates using the Benjamini-Hochberg false discovery rate (FDR) correction. Associations with FDR-adjusted *p*-values < 0.1 were considered statistically significant, while those with nominal *p*-values < 0.05 but FDR ≥ 0.1 were regarded as suggestive evidence [47].

### 2.5 Mediation Analysis

Selected immune cells, gut microbiota, inflammatory proteins, plasma lipids, and metabolites showed the following: first, significant causal associations with IPF, with no evidence of heterogeneity or pleiotropy among these factors; second, the effects of immune cells on candidate mediators were assessed using identical selection criteria. Third, conducted two-step MR analysis examining: the total effect of immune cells on IPF (βall), the effect of immune cells on potential mediators (including gut microbiota, inflammatory protein, plasma lipidome, and metabolites) (β1), and the effect of these mediators on IPF (β2). For positive βall: β1 and β2 must share direction (both positive/negative) [48]. Conversely, for negative βall: β1 and β2 must have opposing signs. Subsequently, the mediation effect was calculated using the product of coefficients method (β1×β2), and the proportion mediated was estimated as the ratio of the mediation effect to the total effect: (β1×β2) / βall. The 95% confidence interval (CI) for the mediation effect was determined using the Delta method [49]. Additionally, the direct effect was obtained by subtracting the mediation effect from the total effect: (βall – β1×β2). Mediation effects with *P*<0.05 were considered statistically significant.

### 2.6 Enrichment Analysis

We first identified SNPs significantly associated with both immune cells and IPF risk, then annotated them using g:Profiler (https://biit.cs.ut.ee/gprofiler/snpense), a versatile bioinformatics resource that enables SNP-to-gene mapping, functional enrichment analysis, and cross-species ortholog alignment [50]. Subsequent functional analyses of corresponding genes were performed *via* the Bioinformatics Platform (https://www.bioinformatics.com.cn/) using R packages, including clusterProfiler for gene ontology (GO) covering Biological Process, Molecular Function, Cellular Component, and Kyoto encyclopedia of genes and genomes (KEGG) pathway analyses (*P* < 0.05), org.Hs.eg.db for annotation, and ggplot2 and ComplexHeatmap for visualization.

## 3 RESULTS

### 3.1 Causal Effects of Immune Cells on IPF

Through MR analysis employing the IVW method, we systematically investigated causal associations between 731 immune cell phenotypes and IPF risk. Our findings identified nine immunophenotypes demonstrating significant causal relationships with IPF, comprising eight protective factors and one risk factor. Notably, three CD39+ CD8br Treg subsets including absolute counts (AC), percentage of CD8br Tregs (%CD8br Treg), and percentage of total T cells (%T cell) - exhibited consistent protective effects, with odds ratios ranging from 0.807 (95% CI: 0.726-0.898, *P*<0.0001) to 0.819 (95% CI: 0.736-0.910, *P*=0.0002). Conversely, elevated levels of Activated Treg %CD4 Treg were associated with increased IPF risk (OR=1.107, 95% CI: 1.009-1.214, *P*=0.0310) (Fig. **2A**).

The robustness of these associations was comprehensively validated through multiple lines of evidence, including consistent directional effects across four complementary MR methods (Supplementary Fig. **1**), nonsignificant heterogeneity and pleiotropy in sensitivity analyses (Tables **S1-S2**), and null findings in reverse MR analyses examining IPF's effect on immunophenotypes (Fig. **2B**). This multilayered validation strongly supports the reliability of our causal inferences about immune regulation in IPF pathogenesis.

### 3.2 Causal Effects of Gut Microbiota on IPF

We applied the IVW method to assess causal associations between 473 gut microbiota taxa and IPF risk, and sensitivity analyses indicated that the results were not materially affected by heterogeneity or horizontal pleiotropy.

Bidirectional MR analysis revealed 9 significant microbiota-IPF associations, including 4 protective taxa and 5 risk-enhancing taxa. Protective effects were observed for *Phylum SAR324* (OR=0.261, 95% CI: 0.093-0.732, *P*=0.0107), *Species Rubneribacter sp002159915* (OR=0.393, 95% CI: 0.235-0.657, *P*=0.0004), and *Genus Phocea* (OR=0.613, 95% CI: 0.393-0.958, *P*=0.0318). Conversely, *Phylum Paceibacteria* (OR=13.752, 95% CI: 1.853-102.077, *P*=0.0104), *Class Thermococci* (OR=2.628, 95% CI: 1.028-6.717, *P*=0.0436), and *Species GCA-900199385 sp900320755* (OR=2.260, 95% CI: 1.157-4.414, *P*=0.0170) demonstrated elevated IPF risk (Fig. **3A**).

These associations showed consistent directionality across four alternative MR methods (Supplementary Fig. **2**) with no evidence of heterogeneity or pleiotropy (Tables **S3-S4**). The absence of reverse causation (Fig. **3B**) strengthened evidence for unidirectional microbiota-to-IPF effects.

### 3.3 Causal Effects of Inflammatory Proteins on IPF

Forward MR analysis revealed three inflammatory proteins exhibiting protective associations with IPF risk (Fig. **4A**). Specifically, elevated levels of leukemia inhibitory factor receptor (LIFR, OR=0.770, 95% CI: 0.635-0.935, *P*=0.0084), tumor necrosis factor superfamily member 12 (TNFSF12, OR=0.796, 95% CI: 0.636-0.995, *P*=0.0447), and C-C motif chemokine ligand 25 (CCL25, OR=0.918, 95% CI: 0.847-0.994, *P*=0.0361) were inversely related to IPF development. These protective effects remained consistent across four alternative MR methods (Supplementary Fig. **3**), with sensitivity analyses showing no substantial heterogeneity or horizontal pleiotropy (Tables **S5-S6**).

Reverse MR analysis further demonstrated no significant causal effects of IPF on these inflammatory markers (Fig. **4B**), supporting the unidirectional nature of these protective relationships.

### 3.4 Causal Effects of Plasma Lipidome on IPF

Forward MR analysis identified sterol ester (27:1/18:3) as a risk factor for IPF (OR=1.223, 95% CI: 1.007-1.486, *P*=0.0422), with elevated levels associated with increased disease susceptibility (Fig. **5A**). This association remained consistent across four complementary MR approaches (Supplementary Fig. **4**), while sensitivity analyses confirmed the absence of significant heterogeneity or horizontal pleiotropy (Tables **S7-S8**).

Reverse MR analysis showed no evidence of IPF influencing sterol ester (27:1/18:3) levels (Fig. **5B**), supporting a unidirectional causal relationship.

### 3.5 Causal effects of Plasma Metabolites on IPF

Our IVW-based MR analysis revealed 29 plasma metabolites showing significant causal associations with IPF, comprising 13 protective and 16 risk-associated metabolites. Protective effects were observed for 1-oleoylglycerol (18:1) (OR=0.663, 95% CI: 0.501-0.876, *P*=0.0039), stearoylcarnitine (OR=0.712, 95% CI: 0.545-0.930, *P*=0.0128), and isovalerylcarnitine (C5) (OR=0.733, 95% CI: 0.559-0.960, *P*=0.0240). Conversely, elevated levels of X-11308 (OR=1.479, 95% CI: 1.045-2.095, *P*=0.0273), gamma-glutamyl-2-aminobutyrate (OR=1.472, 95% CI: 1.019-2.126, *P*=0.0391), and arachidoylcarnitine (C20) (OR=1.370, 95% CI: 1.080-1.739, *P*=0.0095) were associated with increased IPF risk (Fig. **6A**).

These associations were consistently replicated across four alternative MR methods (Supplementary Fig. **5**) and remained robust in sensitivity analyses, with no evidence of heterogeneity or horizontal pleiotropy (Tables **S9-S10**). Reverse MR analysis showed no significant influence of IPF on metabolite levels (Fig. **6B**), confirming the unidirectional nature of these relationships.

### 3.6 Mediation Analysis

To elucidate the potential mediation pathways underlying the immune-IPF relationship, we performed a comprehensive two-step MR analysis. After rigorous selection and sensitivity checks (Tables **S11-S12**), we identified several significant mediation pathways involving gut microbiota, inflammatory proteins, and plasma metabolites (Fig. **7**, Table **1**).

Notably, the detrimental effect of Activated Treg% CD4 Treg on IPF risk was significantly mediated by the reduction of *Phylum SAR324* (mediated proportion= 16.8%, β=0.0171, *p*=0.0267), TNFSF12 (mediated proportion=10.8%, β=0.0109, *P*=0.0321), and CCL25 (mediated proportion=4.94%, β=0.00501, *P*=0.0369).

Conversely, the protective effect of HLA DR on CD33-HLA DR+ Myeloid cells was partly conveyed through increasing the levels of LIFR (mediated proportion=22.1%, β=-0.0154, *P*=0.0085) and decadienedioic acid (C10:2-DC) (mediated proportion=8.38%, β=-0.00583, *P*=0.0168). However, this protective effect was also partially counteracted by an increase in arachidoylcarnitine (C20) (mediated proportion = -19%, β = 0.0132, *P* = 0.0285).

Furthermore, the protective role of CD39+ CD4+ %T cells was mediated by suppressing the abundance of *Species CHKCI006 sp900018345* (mediated proportion=14.8%, β=-0.0137, *P*=0.0444). Meanwhile, the beneficial effect of CD39+ CD8br Treg % CD8br Treg was partially explained by its positive effect on the metabolite X-16935 (mediated proportion=5.68%; β=-0.012, *P*=0.0456).

Additionally, we observed that CD39+ CD4+ AC Treg influenced IPF risk through LIFR (mediated proportion=-15.6%, β=0.0148, *P*=0.0232), and Activated Treg%CD4 Treg also exerted part of its effect *via* isobutyrylcarnitine (C4) (mediated proportion=-7.07%, β=-0.00717, *P*=0.0354).

Negative mediated proportions observed across several pathways indicate suppressive mediation. This occurs when the mediator's effect opposes the exposure's total effect. For example, a negative proportion can occur when a risk factor exerts its effect by suppressing a protective mediator, again creating sign inconsistency. Thus, these negative values do not indicate an error in calculation but rather imply complex, counterintuitive biological mechanisms in which the mediator variable suppresses or masks the true strength of the relationship between the exposure and the outcome.

### 3.7 Results of Enrichment Analysis

Following the conversion of immune cell- and IPF-associated SNPs into corresponding genes, we conducted comprehensive enrichment analyses (Fig. **8**, Table **S13**). Key functional clusters emerging from this analysis encompassed developmental growth inhibition, antigen receptor-mediated signal transduction, growth suppression regulation, and guanyl-nucleotide exchange factor activity (Fig. **8A**). KEGG pathway analysis further demonstrated that immune cell genetic effects on IPF pathogenesis were predominantly mediated through Epstein-Barr virus infection, galactose metabolic processes, asthma-related pathways, primary immunodeficiency mechanisms, and IgA-associated intestinal immune network regulation (Fig. **8B**). The genomic distribution of these significant genes is visually presented in Fig. (**8C**).

## 4 DISCUSSION

Emerging evidence highlights the complex interplay between immune dysregulation and IPF progression, mediated by gut-lung axis interactions, inflammatory protein signaling, and systemic metabolic reprogramming [51-55]. Dysbiosis of the gut microbiota exacerbates IPF by promoting pulmonary interleukin 17B (IL-17B) production from alveolar macrophages, which drives neutrophil recruitment and T helper 17 (Th17) cell differentiation, amplifying fibrotic responses [56]. Concurrently, inflammatory proteins such as TGF-β and platelet-derived growth factor (PDGF) activate pro-fibrotic macrophages and fibroblasts, while dysregulated plasma metabolites impair immune homeostasis, further accelerating ECM deposition [57]. Notably, plasma lipidomic shifts correlate with IPF severity, potentially modulating immune cell function through membrane receptor signaling or lipid raft formation [58]. Conversely, short-chain fatty acids (SCFAs) from commensal gut bacteria may attenuate fibrosis by promoting anti-inflammatory Tregs and suppressing the Interleukin 6 (IL-6)/signal transducer and activator of transcription 3 (STAT3) pathways [59-62]. Targeting these multi-omics hubs represents a promising therapeutic strategy to rebalance immune-fibrotic crosstalk in IPF.

Fibrosis is characterized by excessive ECM deposition, driven by dysregulated interactions between immune cells and stromal components [63, 64]. Immune cells, particularly macrophages and T cell subsets, exhibit functional plasticity in fibrotic niches, orchestrating both pro-fibrotic and resolution phases through cytokine networks and direct cellular crosstalk [65-67]. In the present study, using bidirectional MR analysis alongside mediation analysis, we revealed key insights. Our study revealed genetic evidence linking 9 immune cell phenotypes, 9 gut microbiota taxa, 3 inflammatory proteins, 1 lipid species, and 29 plasma metabolites with IPF risk, suggesting they may contribute to disease mechanisms. Moreover, we demonstrated that immune cells, including Activated CD4 Treg, HLA DR+ CD33- Myeloid cell, CD39+ CD4+ T cell, and CD39+ CD8br Treg, likely participate in the modulation of IPF by several phenotypes of gut microbiota, inflammatory proteins, and plasma metabolites. Additionally, we identified 12 immune cell-associated genes closely linked to the pathological mechanisms of IPF and performed GO and KEGG enrichment analyses along with chromosomal localization, laying the foundation for our further research.

To our knowledge, this is the first evidence that Activated CD4 Treg exacerbates IPF progression by suppressing protective mechanisms mediated by the Phyla *SAR324*, CCL25, and TNFSF12.​​ Recent studies reveal that activated Tregs, while traditionally immunosuppressive, may paradoxically promote fibrogenesis by inhibiting microbial and immunomodulatory pathways. *SAR324*, a deep-sea bacterial phylum, has been implicated in carbon fixation and immune homeostasis, but its suppression by Tregs may disrupt lung microbial balance, exacerbating fibrosis. Further studies are required in order to elucidate the mechanism by which activated Tregs suppress SAR324 in pulmonary fibrosis [68-71]. CCL25, a chemokine critical for gut-lung immune crosstalk, is downregulated by Tregs *via* CCR9 inhibition, impairing anti-fibrotic lymphocyte recruitment [72, 73]. Similarly, TNFSF12, a tumor necrosis factor superfamily member with tissue-repair functions, is suppressed by Tregs *via* signal transducer and activator of transcription 5 (STAT5)-dependent pathways, further accelerating ECM deposition [74]. These findings highlight a dual role for Tregs in IPF, in which their overactivation may counteract endogenous protective signals. Notably, given that *Phylum SAR324*, CCL25, and TNFSF12 all mediate the regulatory effects of Activated CD4 Treg on IPF, and their protective effects against IPF are consistently suppressed by Activated CD4 Treg, we hypothesize that there may exist a synergistic mechanism whereby Activated CD4 Treg simultaneously inhibits these three protective factors to promote IPF progression. However, this hypothesis warrants further experimental validation through additional biological studies.

It is worth noting that HLA DR+ CD33- Myeloid cells appear to attenuate IPF progression by potentiating the anti-oxidative effects of C10:2-DC *via* PPAR-γ pathways [75]. CD39+ CD4+ T cells and CD39+ CD8br Treg exhibit differential immunomodulatory effects in IPF pathogenesis. Specifically, CD39+ CD4+ T cells demonstrate protective properties by suppressing the profibrotic factor CHKCI006 sp900018345, while CD39+ CD8+ Treg cells promote the anti-fibrotic factor X-16935, collectively contributing to IPF protection. These coordinated actions likely suggest several novel immunometabolic axis in IPF pathogenesis that merits further exploration. However, our findings demonstrate that liposomes do not directly mediate immune cell involvement in IPF pathogenesis, suggesting that liposomes may regulate IPF progression through alternative indirect mechanisms.

The methodical filtering steps, consistent with established MR protocols, yielded a refined set of robust IVs for genetic association analysis, thereby upholding the study's methodological rigor and reproducibility. To elucidate the mechanistic links between immune cells and IPF pathogenesis, we analyzed SNPs demonstrating significant associations with both immune phenotypes and IPF risk. Functional enrichment analyses (GO/KEGG) of these genetic variants highlighted their involvement in immune regulatory pathways, reinforcing the crucial role of immune dysregulation in IPF development.

While these findings provide valuable insights, certain limitations warrant consideration. All GWAS datasets are of European ancestry, which may limit generalizability to other populations; Some metabolites (*e.g.*, X-16935) remain poorly characterized, reducing biological interpretability. Notably, the IPF, immune cell, gut microbiota, inflammatory protein, plasma lipidome, and metabolite data were derived from distinct, non-overlapping cohorts, thereby reducing the risk of overlap bias in our findings. ​​However, this design may also introduce inconsistencies across studies due to differences in populations, data collection methods, or statistical power.

Although our MR analysis identified several robust and statistically significant causal associations, it is essential to note that the estimated effect sizes for some of these relationships were modest. This suggests that while these microbial features may play a role in IPF pathogenesis, their individual clinical utility as standalone biomarkers or therapeutic targets might be limited. The strength of this study lies not in the magnitude of individual effects, but in the integrative multi-omics approach that systematically unravels the complex, interconnected pathways of the gut-lung axis. Future research combining larger-scale genomic data with clinical phenotypes is needed to validate these associations further and assess their potential translational relevance.

Although MR offers robust causal inference, potential pleiotropy and unmeasured environmental factors may still influence results, despite our comprehensive sensitivity analyses. Subsequent investigations should combine population-based validation with experimental approaches to fully characterize the identified biological mechanisms.

## CONCLUSION

Through comprehensive MR analysis, this study systematically characterized causal relationships and mediation pathways connecting immune cells, gut microbiota, inflammatory proteins, plasma lipids, and metabolites in IPF pathogenesis. These findings highlight potential therapeutic targets, particularly within gut microbial communities, inflammatory mediators, metabolic networks, and key immune regulators. While providing mechanistic insights into IPF development, these observations require experimental confirmation to establish definitive causal links.

## Figures and Tables

**Fig. (1):**
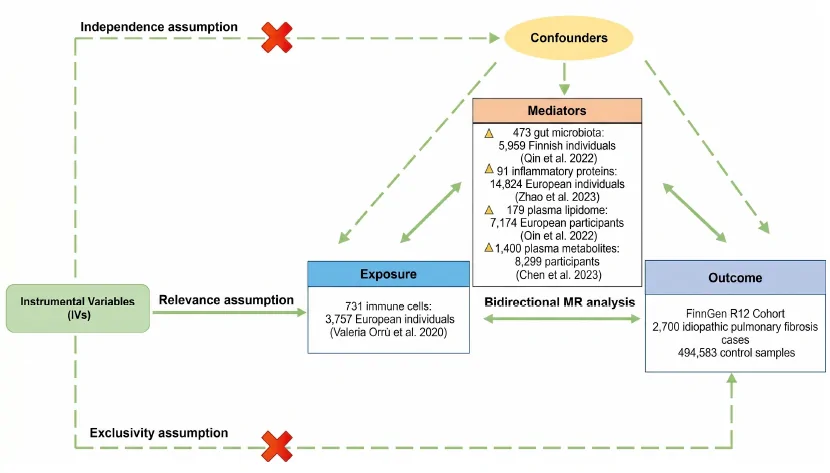
Study design overview. Relevance: IVs show a significant association with exposure. Independence: IVs must remain unconfounded by exposure-outcome covariates. Exclusivity: IVs influence outcome exclusively through exposure pathways.

**Fig. (2):**
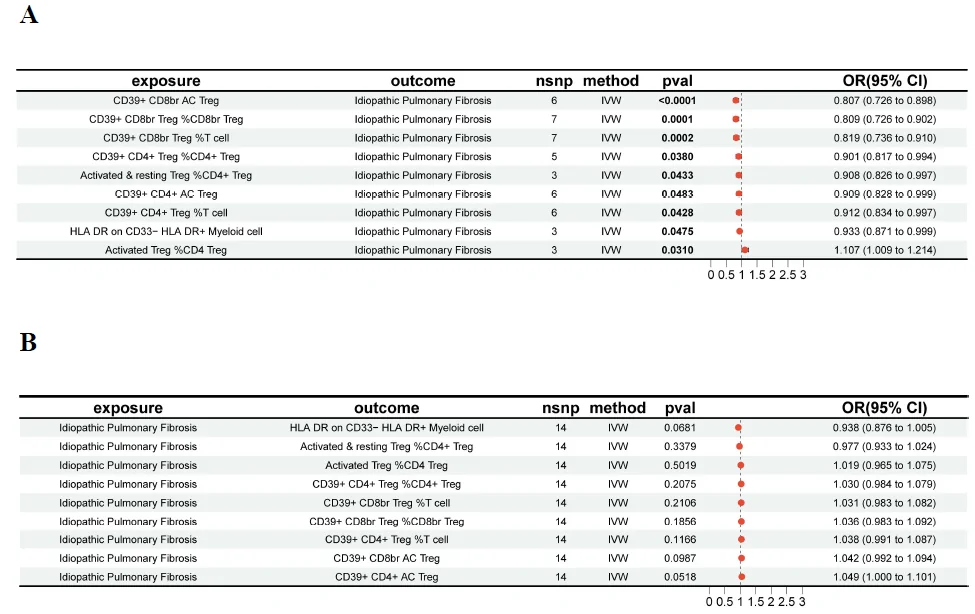
MR analysis of immune cells and IPF. (**A**) Causal effects of immune cell phenotypes on IPF risk. (**B**) Reverse MR analysis assessing effects of IPF on immune cell phenotypes. The odds ratios (ORs), 95% confidence intervals (CIs), and *P*-values were calculated for each Mendelian randomization analysis method. A *P*-value < 0.05 was considered statistically significant. nsnp: number of single nucleotide polymorphisms; IVW: inverse-variance weighted.

**Fig. (3):**
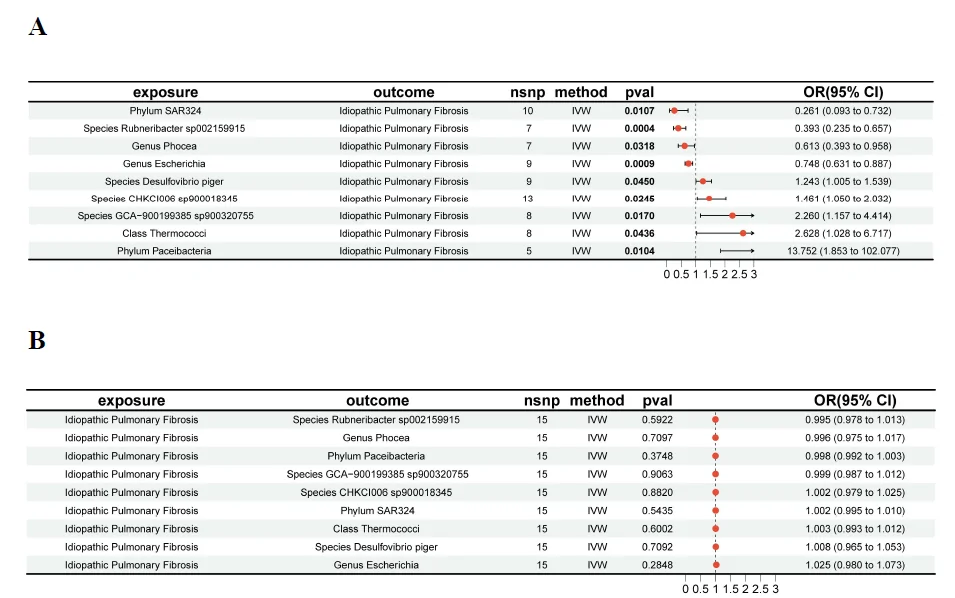
MR analysis of gut microbiota and IPF. (**A**) Causal effects of gut microbiota on IPF risk. (**B**) Reverse MR analysis assessing effects of IPF on gut microbiota. The odds ratios (ORs), 95% confidence intervals (CIs), and *P*-values were calculated for each Mendelian randomization analysis method. A *P*-value < 0.05 was considered statistically significant. nsnp: number of single nucleotide polymorphisms; IVW: inverse-variance weighted.

**Fig. (4):**
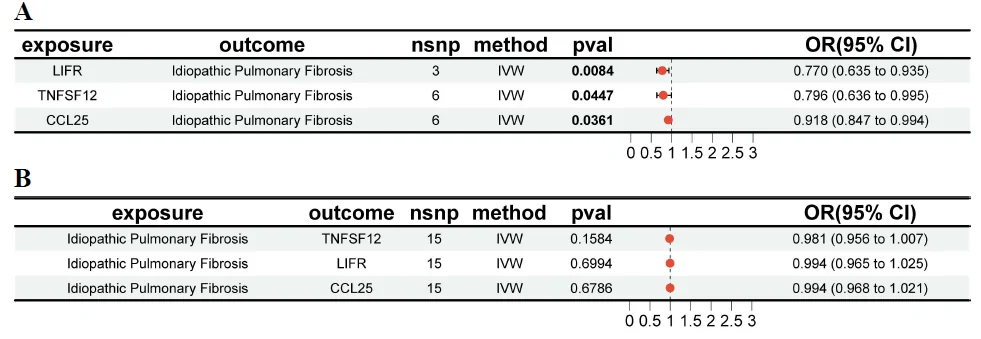
MR analysis of inflammatory proteins and IPF. (**A**) Causal effects of inflammatory proteins on IPF risk. (**B**) Reverse MR analysis assessing effects of IPF on inflammatory proteins. The odds ratios (ORs), 95% confidence intervals (CIs), and *P*-values were calculated for each Mendelian randomization analysis method. A *P*-value 0.05 was considered statistically significant. nsnp: number of single nucleotide polymorphisms; IVW: inverse-variance weighted.

**Fig. (5):**
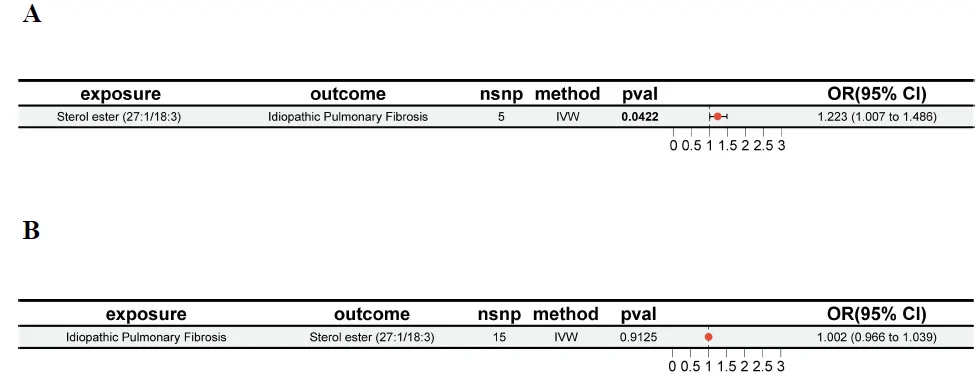
MR analysis of plasma lipidome and IPF. (**A**) Causal effects of plasma lipidome on IPF risk. (**B**) Reverse MR analysis assessing effects of IPF on plasma lipidome. The odds ratios (ORs), 95% confidence intervals (CIs), and *P*-values were calculated for each Mendelian randomization analysis method. A *P*-value 0.05 was considered statistically significant. nsnp: number of single nucleotide polymorphisms; IVW: inverse-variance weighted.

**Fig. (6):**
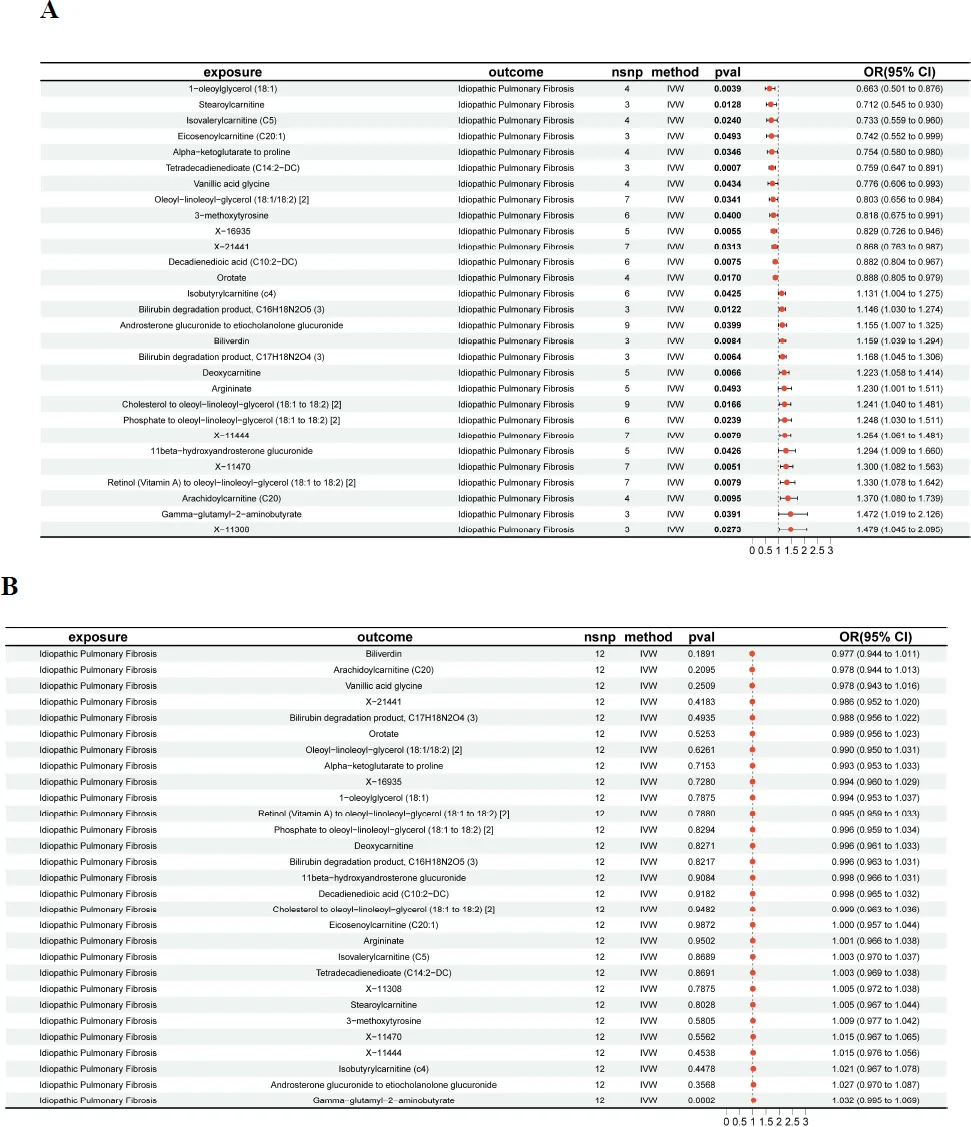
MR analysis of plasma metabolites and IPF. (**A**) Causal effects of plasma metabolites on IPF risk. (**B**) Reverse MR analysis assessing effects of IPF on plasma metabolites. The odds ratios (ORs), 95% confidence intervals (CIs), and *P*-values were calculated for each Mendelian randomization analysis method. A *P*-value 0.05 was considered statistically significant. nsnp: number of single nucleotide polymorphisms; IVW: inverse-variance weighted.

**Fig. (7):**
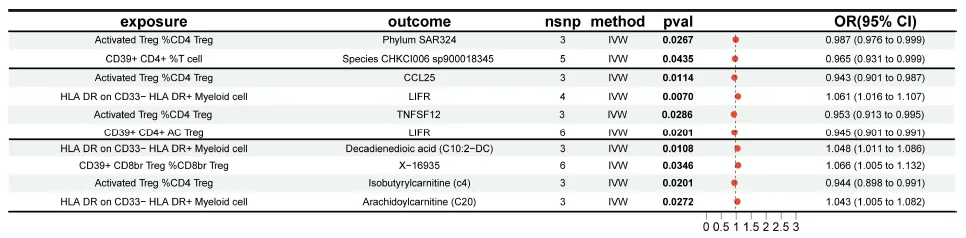
The results of the mediation analysis. The odds ratios (ORs), 95% confidence intervals (CIs), and *P*-values were calculated for each Mendelian randomization analysis method. A *P*-value 0.05 was considered statistically significant. nsnp: number of single-nucleotide polymorphisms; IVW: inverse-variance weighted.

**Fig. (8):**
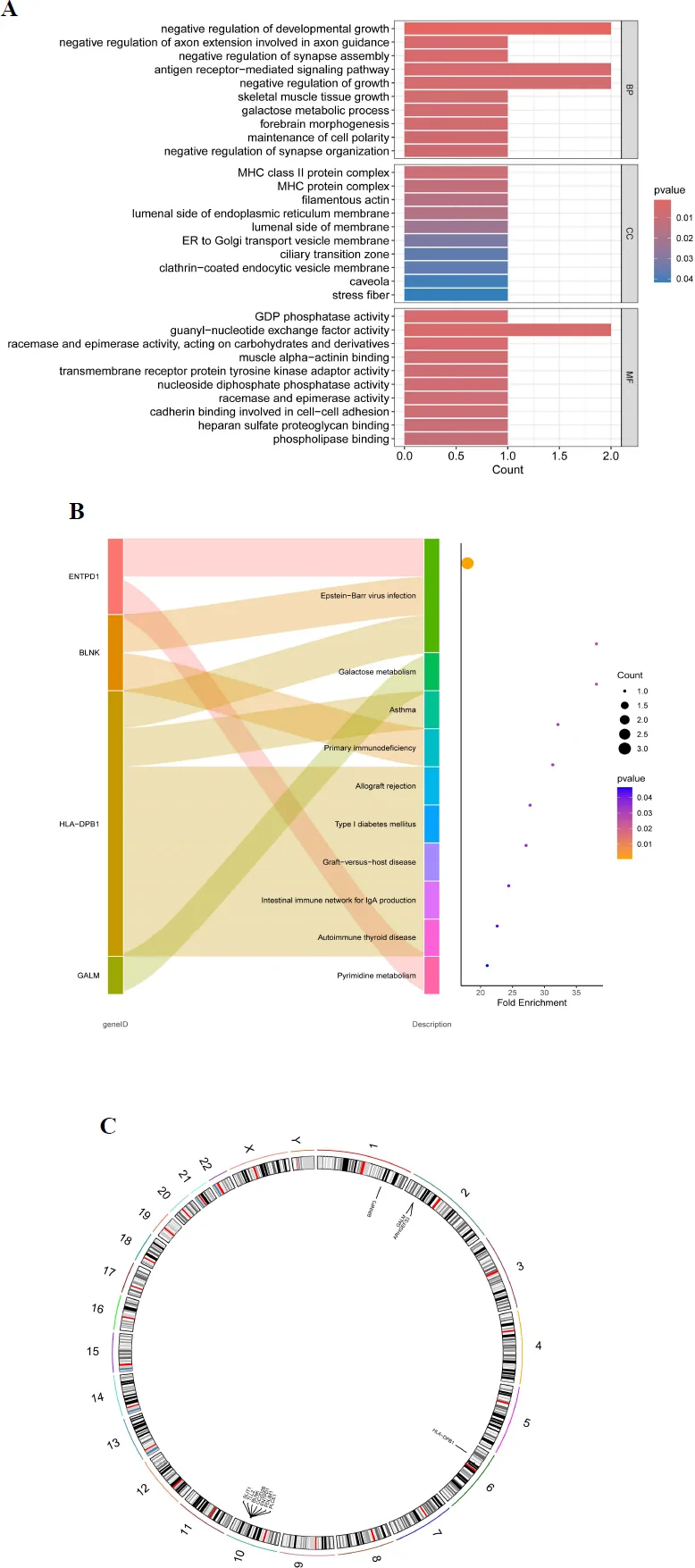
Enrichment analysis. (**A**) GO analysis, including biological processes, molecular functions (MFs), and cellular components. (**B**) KEGG pathway enrichment analysis. (**C**) Chromosomal locations of significant genes are displayed in a circular plot.

**Table 1:** Mediation effects and proportions of each mediator.

**Exposure** **(Immune Cells)**	**Mediator** **(Gut Microbiota)**	**Outcome**	**Mediated Effect**	**Mediated Proportion**	**Direct Effect**	***P*-value**
Activated Treg %CD4 Treg	*Phylum SAR324*	IPF	0.0171 (0.00197, 0.0321)	16.8% (1.94%, 31.7%)	0.084	0.0267
CD39+ CD4+ %T cell	*Species CHKCI006 sp900018345*	IPF	-0.0137 (-0.027, -0.00034)	14.8% (29.2%, 0.368%)	-0.079	0.0444
Activated Treg %CD4 Treg	CCL25	IPF	0.00501 (0.000304, 0.00972)	4.94% (0.3%, 9.58%)	0.096	0.0369
HLA DR on CD33- HLA DR+ Myeloid cell	LIFR	IPF	-0.0154 (-0.0268, -0.00392)	22.1% (38.5%, 5.64%)	-0.054	0.0085
Activated Treg %CD4 Treg	TNFSF12	IPF	0.0109 (0.000934, 0.0209)	10.8% (0.92%, 20.6%)	0.091	0.0321
CD39+ CD4+ AC Treg	LIFR	IPF	0.0148 (0.00202, 0.0275)	-15.6% (-2.13%, -29%)	-0.110	0.0232
HLA DR on CD33- HLA DR+ Myeloid cell	Decadienedioic acid (C10:2-DC)	IPF	-0.00583 (-0.0106, -0.00105)	8.38% (15.3%, 1.51%)	-0.064	0.0168
CD39+ CD8br Treg %CD8br Treg	X-16935	IPF	-0.012 (-0.0238, -0.000234)	5.68% (11.2%, 0.11%)	-0.200	0.0456
Activated Treg %CD4 Treg	Isobutyrylcarnitine (c4)	IPF	-0.00717 (-0.0139, -0.000491)	-7.07% (-13.7%, -0.484%)	0.109	0.0354
HLA DR on CD33- HLA DR+ Myeloid cell	Arachidoylcarnitine (C20)	IPF	0.0132 (0.00139, 0.0251)	-19% (-2%, -36%)	-0.083	0.0285

## Data Availability

This study utilized only publicly available data, with details on data sources and processing provided in the Materials and Methods section and the online supplementary information. Additional information can be obtained from the corresponding author upon request.

## LIST OF ABBREVIATIONS

β: Effect Sizes
CCL25: C-C motif chemokine Ligand 25
CI: Confidence Interval
EA: Effect Allele
EAF: Effect Allele Frequency
ECM: Extracellular Matrix
FDR: False Discovery Rate
GO: Gene Ontology
GWAS: Genome-Wide Association Studies
IL-6: Interleukin 6
IL-17B: Interleukin 17B
IPF: Idiopathic Pulmonary Fibrosis
IVs: Instrumental Variables
IVW: Inverse Variance Weighted
KEGG: Kyoto Encyclopedia of Genes and Genomes
LIFR: Leukemia Inhibitory Factor Receptor
LD: Linkage Disequilibrium
MFs: Molecular Functions
MFI: Median Fluorescence Intensity
MR: Mendelian Randomization
OA: Other Allele
OR: Odds Ratio
PDGF: Platelet-Derived Growth Factor
RCTs: Randomized Controlled Trials
SALs: Surface Antigen Levels
SCFAs: Short-Chain Fatty Acids
SE: Standard Error
SNPs: Single Nucleotide Polymorphisms
STAT3: Signal Transducer and Activator of Transcription 3
STAT5: Signal Transducer and Activator of Transcription 5
STROBE-MR: Strengthening the Reporting of Observational studies in Epidemiology using Mendelian Randomization
TGF-β: Transforming Growth Factor-β
Th17 Cells: T Helper 17 Cells
TNFSF12: Tumor Necrosis Factor Superfamily member 12
Treg: Regulatory T Cell

## STANDARDS OF REPORTING

STROBE guidelines were followed.
