# Supplementary material for: Immune-Mediated Gut-Lung Axis in IPF: Bidirectional Mendelian Randomization and Mediation Analysis of Microbiota, Inflammatory Proteins, and Metabolites
Source: Endocr Metab Immune Disord Drug Targets. 2026 Jun 3;26:E18715303416212. doi: 10.2174/0118715303416212251126093242 (PMC13670199; doi:10.2174/0118715303416212251126093242)
Supplement: Supplementary file 1 [file EMIDDT-26-E18715303416212_SD1.pdf]

Supplementary Material

Immune-Mediated Gut-Lung Axis in IPF: Bidirectional Mendelian Randomization and Mediation Analysis of Microbiota, Inflammatory Proteins, and Metabolites

Jiaxing Zhao[1,2,#], Zenghui Liu[1,2,#], Lu Kuang[1], Aijia Dong[2], Heyue Gong[2], Huayu Yin[2], Shaoguo Wu[1], Dabin Liu[1], Xuehui Liu[1], Shuyan Liu[1] and Limei Wu[1,2,*]

[1]*Department of Laboratory Medicine, the Affiliated Guangzhou Twelfth People's Hospital, Guangzhou Medical University, Guangzhou, Guangdong, China;* [2]*Department of Immunology, Mudanjiang Medical University, Mudanjiang, Heilongjiang, China*

exposure	outcome	nsnp	method	pval	OR(95% CI)
Activated & resting Treg %CD4+ Treg	Idiopathic Pulmonary Fibrosis	3	MR Egger	0.4744	0.920 (0.792 to 1.069)
		3	Weighted median	**0.0364**	0.903 (0.820 to 0.994)
		3	Inverse variance weighted	**0.0433**	0.908 (0.826 to 0.997)
		3	Simple mode	0.2157	0.869 (0.745 to 1.014)
		3	Weighted mode	0.1644	0.903 (0.823 to 0.991)
Activated Treg %CD4 Treg	Idiopathic Pulmonary Fibrosis	3	MR Egger	0.4776	1.114 (0.914 to 1.358)
		3	Weighted median	**0.0489**	1.106 (1.000 to 1.223)
		3	Inverse variance weighted	**0.0310**	1.107 (1.009 to 1.214)
		3	Simple mode	0.2632	1.097 (0.975 to 1.235)
		3	Weighted mode	0.2247	1.114 (0.986 to 1.257)
CD39+ CD4+ Treg %CD4+ Treg	Idiopathic Pulmonary Fibrosis	5	MR Egger	0.6847	0.916 (0.625 to 1.344)
		5	Weighted median	0.1091	0.910 (0.810 to 1.021)
		5	Inverse variance weighted	**0.0380**	0.901 (0.817 to 0.994)
		5	Simple mode	0.2202	0.884 (0.748 to 1.044)
		5	Weighted mode	0.2796	0.927 (0.823 to 1.044)
CD39+ CD4+ Treg %T cell	Idiopathic Pulmonary Fibrosis	6	MR Egger	0.8258	0.962 (0.698 to 1.327)
		6	Weighted median	0.4698	0.960 (0.859 to 1.073)
		6	Inverse variance weighted	**0.0428**	0.912 (0.834 to 0.997)
		6	Simple mode	0.3114	0.921 (0.799 to 1.063)
		6	Weighted mode	0.4196	0.949 (0.844 to 1.067)
CD39+ CD4+ AC Treg	Idiopathic Pulmonary Fibrosis	6	MR Egger	0.5864	0.906 (0.652 to 1.258)
		6	Weighted median	0.1191	0.913 (0.814 to 1.024)
		6	Inverse variance weighted	**0.0483**	0.909 (0.828 to 0.999)
		6	Simple mode	0.2329	0.887 (0.747 to 1.055)
		6	Weighted mode	0.2396	0.923 (0.820 to 1.038)
CD39+ CD8br Treg %CD8br Treg	Idiopathic Pulmonary Fibrosis	7	MR Egger	0.0705	0.765 (0.609 to 0.962)
		7	Weighted median	**0.0078**	0.833 (0.728 to 0.953)
		7	Inverse variance weighted	**0.0001**	0.809 (0.726 to 0.902)
		7	Simple mode	0.0802	0.835 (0.707 to 0.988)
		7	Weighted mode	**0.0406**	0.834 (0.727 to 0.956)
CD39+ CD8br Treg %T cell	Idiopathic Pulmonary Fibrosis	7	MR Egger	0.1510	0.822 (0.654 to 1.031)
		7	Weighted median	**0.0058**	0.834 (0.733 to 0.949)
		7	Inverse variance weighted	**0.0002**	0.819 (0.736 to 0.910)
		7	Simple mode	0.0962	0.835 (0.698 to 0.999)
		7	Weighted mode	**0.0464**	0.835 (0.725 to 0.962)
CD39+ CD8br AC Treg	Idiopathic Pulmonary Fibrosis	6	MR Egger	0.0993	0.792 (0.639 to 0.981)
		6	Weighted median	**0.0068**	0.838 (0.738 to 0.953)
		6	Inverse variance weighted	**<0.0001**	0.807 (0.726 to 0.898)
		6	Simple mode	0.1017	0.839 (0.706 to 0.996)
		6	Weighted mode	0.0564	0.841 (0.733 to 0.965)
HLA DR on CD33− HLA DR+ Myeloid cell	Idiopathic Pulmonary Fibrosis	3	MR Egger	0.7511	0.942 (0.708 to 1.252)
		3	Weighted median	**0.0403**	0.930 (0.868 to 0.997)
		3	Inverse variance weighted	**0.0475**	0.933 (0.871 to 0.999)
		3	Simple mode	0.2507	0.908 (0.807 to 1.022)
		3	Weighted mode	0.1853	0.931 (0.867 to 0.999)

Fig. (S1).

exposure	outcome	nsnp	method	pval	OR(95% CI)
Species CHKCI006 sp900018345	Idiopathic Pulmonary Fibrosis	13	MR Egger	0.9772	1.013 (0.432 to 2.374)
		13	Weighted median	0.1784	1.363 (0.868 to 2.141)
		13	Inverse variance weighted	**0.0245**	1.461 (1.050 to 2.032)
		13	Simple mode	0.5958	1.216 (0.602 to 2.459)
		13	Weighted mode	0.6250	1.199 (0.589 to 2.440)
Species Desulfovibrio piger	Idiopathic Pulmonary Fibrosis	9	MR Egger	0.1972	1.463 (0.867 to 2.470)
		9	Weighted median	0.2901	1.188 (0.864 to 1.633)
		9	Inverse variance weighted	**0.0450**	1.243 (1.005 to 1.539)
		9	Simple mode	0.8503	1.052 (0.631 to 1.754)
		9	Weighted mode	0.8353	1.056 (0.641 to 1.742)
Genus Escherichia	Idiopathic Pulmonary Fibrosis	9	MR Egger	0.2796	0.765 (0.489 to 1.198)
		9	Weighted median	**0.0108**	0.753 (0.605 to 0.937)
		9	Inverse variance weighted	**0.0009**	0.748 (0.631 to 0.887)
		9	Simple mode	0.1426	0.765 (0.553 to 1.057)
		9	Weighted mode	0.1218	0.760 (0.558 to 1.037)
Species GCA−900199385 sp900320755	Idiopathic Pulmonary Fibrosis	8	MR Egger	0.3469	2.096 (0.506 to 8.688)
		8	Weighted median	0.2211	1.783 (0.706 to 4.500)
		8	Inverse variance weighted	**0.0170**	2.260 (1.157 to 4.414)
		8	Simple mode	0.6288	1.434 (0.354 to 5.808)
		8	Weighted mode	0.6297	1.421 (0.362 to 5.574)
Phylum Paceibacteria	Idiopathic Pulmonary Fibrosis	5	MR Egger	0.5440	7.498 (0.023 to 2444.418)
		5	Weighted median	0.1868	5.834 (0.425 to 80.051)
		5	Inverse variance weighted	**0.0104**	13.752 (1.853 to 102.077)
		5	Simple mode	0.5644	3.261 (0.081 to 130.919)
		5	Weighted mode	0.5480	3.314 (0.092 to 119.271)
Genus Phocea	Idiopathic Pulmonary Fibrosis	7	MR Egger	0.4945	0.724 (0.306 to 1.711)
		7	Weighted median	**0.0471**	0.559 (0.314 to 0.993)
		7	Inverse variance weighted	**0.0318**	0.613 (0.393 to 0.958)
		7	Simple mode	0.1726	0.524 (0.231 to 1.188)
		7	Weighted mode	0.1323	0.511 (0.240 to 1.088)
Species Rubneribacter sp002159915	Idiopathic Pulmonary Fibrosis	7	MR Egger	0.3714	0.513 (0.135 to 1.947)
		7	Weighted median	**0.0078**	0.399 (0.203 to 0.785)
		7	Inverse variance weighted	**0.0004**	0.393 (0.235 to 0.657)
		7	Simple mode	0.0844	0.402 (0.169 to 0.954)
		7	Weighted mode	0.0957	0.393 (0.156 to 0.993)
Phylum SAR324	Idiopathic Pulmonary Fibrosis	10	MR Egger	0.2540	0.237 (0.024 to 2.354)
		10	Weighted median	**0.0460**	0.233 (0.056 to 0.974)
		10	Inverse variance weighted	**0.0107**	0.261 (0.093 to 0.732)
		10	Simple mode	0.1048	0.124 (0.013 to 1.198)
		10	Weighted mode	0.1720	0.132 (0.009 to 1.915)
Class Thermococci	Idiopathic Pulmonary Fibrosis	8	MR Egger	0.7977	1.698 (0.035 to 81.789)
		8	Weighted median	0.2764	2.025 (0.568 to 7.213)
		8	Inverse variance weighted	**0.0436**	2.628 (1.028 to 6.717)
		8	Simple mode	0.3882	2.218 (0.406 to 12.110)
		8	Weighted mode	0.4200	2.125 (0.379 to 11.912)

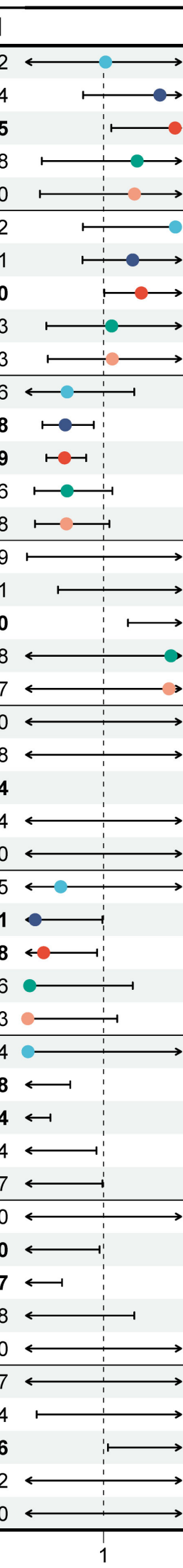

Fig. (S2).

exposure	outcome	nsnp	method	pval	OR(95% CI)
CCL25	Idiopathic Pulmonary Fibrosis	6	MR Egger	0.2420	0.913 (0.802 to 1.040)
		6	Weighted median	0.0718	0.924 (0.848 to 1.007)
		6	Inverse variance weighted	**0.0361**	0.918 (0.847 to 0.994)
		6	Simple mode	0.3202	0.897 (0.739 to 1.088)
		6	Weighted mode	0.1151	0.919 (0.843 to 1.002)
LIFR	Idiopathic Pulmonary Fibrosis	3	MR Egger	0.4434	0.746 (0.461 to 1.206)
		3	Weighted median	**0.0163**	0.782 (0.640 to 0.956)
		3	Inverse variance weighted	**0.0084**	0.770 (0.635 to 0.935)
		3	Simple mode	0.4004	0.851 (0.631 to 1.147)
		3	Weighted mode	0.2260	0.814 (0.644 to 1.028)
TNFSF12	Idiopathic Pulmonary Fibrosis	6	MR Egger	0.4534	0.769 (0.414 to 1.430)
		6	Weighted median	0.1448	0.812 (0.614 to 1.074)
		6	Inverse variance weighted	**0.0447**	0.796 (0.636 to 0.995)
		6	Simple mode	0.3419	0.773 (0.477 to 1.251)
		6	Weighted mode	0.2669	0.822 (0.604 to 1.118)

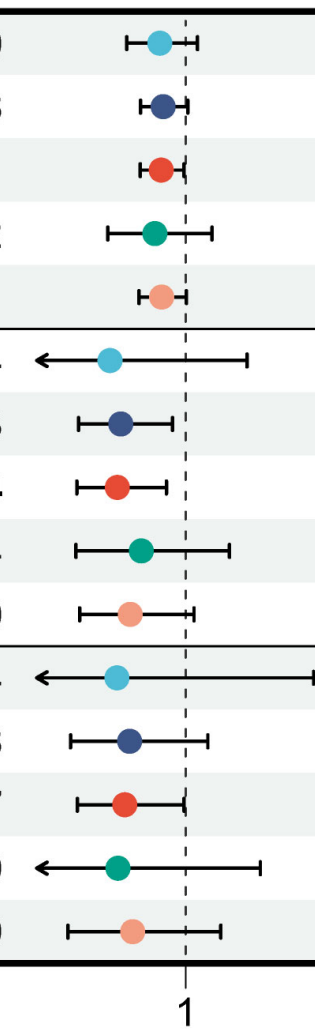

Fig. (S3).

exposure	outcome	nsnp	method	pval	OR(95% CI)
Sterol ester (27:1/18:3)	Idiopathic Pulmonary Fibrosis	5	MR Egger	0.1588	1.644 (0.975 to 2.770)
		5	Weighted median	0.1469	1.192 (0.940 to 1.513)
		5	Inverse variance weighted	**0.0422**	1.223 (1.007 to 1.486)
		5	Simple mode	0.6743	1.080 (0.773 to 1.510)
		5	Weighted mode	0.4154	1.133 (0.865 to 1.482)

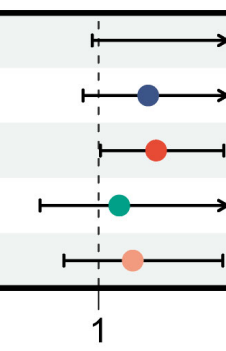

Fig. (S4).

exposure	outcome	nsnp	method	pval	OR(95% CI)
1-oleoylglycerol (18:1)	Idiopathic Pulmonary Fibrosis	4	MR Egger	0.7450	0.760 (0.179 to 3.223)
		4	Weighted median	**0.0134**	0.645 (0.456 to 0.913)
		4	Inverse variance weighted	**0.0039**	0.663 (0.501 to 0.876)
		4	Simple mode	0.1221	0.538 (0.305 to 0.950)
		4	Weighted mode	0.2630	0.733 (0.470 to 1.142)
11beta-hydroxyandrosterone glucuronide	Idiopathic Pulmonary Fibrosis	5	MR Egger	0.1698	1.414 (0.970 to 2.061)
		5	Weighted median	0.0624	1.322 (0.986 to 1.774)
		5	Inverse variance weighted	**0.0426**	1.294 (1.009 to 1.660)
		5	Simple mode	0.3813	1.217 (0.822 to 1.803)
		5	Weighted mode	0.1939	1.306 (0.934 to 1.826)
3-methoxytyrosine	Idiopathic Pulmonary Fibrosis	6	MR Egger	0.2048	0.688 (0.424 to 1.117)
		6	Weighted median	**0.0337**	0.776 (0.614 to 0.981)
		6	Inverse variance weighted	**0.0400**	0.818 (0.675 to 0.991)
		6	Simple mode	0.3327	0.814 (0.559 to 1.186)
		6	Weighted mode	0.0849	0.726 (0.541 to 0.973)
Alpha-ketoglutarate to proline	Idiopathic Pulmonary Fibrosis	4	MR Egger	0.3578	0.707 (0.399 to 1.254)
		4	Weighted median	0.0531	0.730 (0.530 to 1.004)
		4	Inverse variance weighted	**0.0346**	0.754 (0.580 to 0.980)
		4	Simple mode	0.1839	0.679 (0.437 to 1.056)
		4	Weighted mode	0.1238	0.681 (0.478 to 0.971)
Androsterone glucuronide to etiocholanolone glucuronide	Idiopathic Pulmonary Fibrosis	9	MR Egger	0.1218	1.202 (0.979 to 1.476)
		9	Weighted median	0.1618	1.136 (0.950 to 1.358)
		9	Inverse variance weighted	**0.0399**	1.155 (1.007 to 1.325)
		9	Simple mode	0.5231	1.090 (0.846 to 1.405)
		9	Weighted mode	0.2102	1.159 (0.937 to 1.433)
Arachidoylcarnitine (C20)	Idiopathic Pulmonary Fibrosis	4	MR Egger	0.4370	1.377 (0.718 to 2.640)
		4	Weighted median	**0.0295**	1.359 (1.031 to 1.792)
		4	Inverse variance weighted	**0.0095**	1.370 (1.080 to 1.739)
		4	Simple mode	0.2121	1.341 (0.932 to 1.928)
		4	Weighted mode	0.2194	1.334 (0.926 to 1.922)
Argininate	Idiopathic Pulmonary Fibrosis	5	MR Egger	0.6703	1.210 (0.546 to 2.681)
		5	Weighted median	0.0616	1.262 (0.989 to 1.611)
		5	Inverse variance weighted	**0.0493**	1.230 (1.001 to 1.511)
		5	Simple mode	0.1702	1.269 (0.960 to 1.678)
		5	Weighted mode	0.1473	1.266 (0.978 to 1.637)
Bilirubin degradation product, C16H18N2O5 (3)	Idiopathic Pulmonary Fibrosis	3	MR Egger	0.2746	1.209 (1.019 to 1.434)
		3	Weighted median	**0.0088**	1.157 (1.037 to 1.290)
		3	Inverse variance weighted	**0.0122**	1.146 (1.030 to 1.274)
		3	Simple mode	0.3284	1.150 (0.929 to 1.425)
		3	Weighted mode	0.1137	1.157 (1.041 to 1.287)
Bilirubin degradation product, C17H18N2O4 (3)	Idiopathic Pulmonary Fibrosis	3	MR Egger	0.2680	1.236 (1.026 to 1.488)
		3	Weighted median	**0.0056**	1.180 (1.049 to 1.326)
		3	Inverse variance weighted	**0.0064**	1.168 (1.045 to 1.306)
		3	Simple mode	0.2172	1.219 (0.980 to 1.516)
		3	Weighted mode	0.1003	1.181 (1.056 to 1.322)
Biliverdin	Idiopathic Pulmonary Fibrosis	3	MR Egger	0.2851	1.271 (1.014 to 1.592)
		3	Weighted median	**0.0063**	1.170 (1.045 to 1.310)
		3	Inverse variance weighted	**0.0084**	1.159 (1.039 to 1.294)
		3	Simple mode	0.3730	1.136 (0.912 to 1.414)
		3	Weighted mode	0.1148	1.177 (1.045 to 1.326)
Cholesterol to oleoyl-linoleoyl-glycerol (18:1 to 18:2) [2]	Idiopathic Pulmonary Fibrosis	9	MR Egger	0.6130	1.138 (0.705 to 1.838)
		9	Weighted median	0.0870	1.219 (0.972 to 1.529)
		9	Inverse variance weighted	**0.0166**	1.241 (1.040 to 1.481)
		9	Simple mode	0.3984	1.174 (0.825 to 1.671)
		9	Weighted mode	0.2770	1.199 (0.884 to 1.625)
Decadienedioic acid (C10:2-DC)	Idiopathic Pulmonary Fibrosis	6	MR Egger	0.0819	0.803 (0.667 to 0.967)
		6	Weighted median	**0.0050**	0.874 (0.795 to 0.960)
		6	Inverse variance weighted	**0.0075**	0.882 (0.804 to 0.967)
		6	Simple mode	0.2817	0.898 (0.753 to 1.070)
		6	Weighted mode	**0.0458**	0.869 (0.783 to 0.964)
Deoxycarnitine	Idiopathic Pulmonary Fibrosis	5	MR Egger	0.2216	1.260 (0.939 to 1.691)
		5	Weighted median	**0.0047**	1.249 (1.070 to 1.457)
		5	Inverse variance weighted	**0.0066**	1.223 (1.058 to 1.414)
		5	Simple mode	0.3649	1.178 (0.860 to 1.614)
		5	Weighted mode	0.0502	1.273 (1.073 to 1.509)
Eicosenoylcarnitine (C20:1)	Idiopathic Pulmonary Fibrosis	3	MR Egger	0.3935	0.337 (0.074 to 1.534)
		3	Weighted median	0.1155	0.756 (0.534 to 1.071)
		3	Inverse variance weighted	**0.0493**	0.742 (0.552 to 0.999)
		3	Simple mode	0.5430	0.848 (0.544 to 1.323)
		3	Weighted mode	0.4542	0.816 (0.528 to 1.259)
Gamma-glutamyl-2-aminobutyrate	Idiopathic Pulmonary Fibrosis	3	MR Egger	0.4184	1.807 (0.738 to 4.423)
		3	Weighted median	0.0974	1.465 (0.933 to 2.303)
		3	Inverse variance weighted	**0.0391**	1.472 (1.019 to 2.126)
		3	Simple mode	0.2402	1.567 (0.920 to 2.668)
		3	Weighted mode	0.2697	1.453 (0.895 to 2.358)
Isobutyrylcarnitine (c4)	Idiopathic Pulmonary Fibrosis	6	MR Egger	0.0573	1.303 (1.071 to 1.585)
		6	Weighted median	0.1096	1.132 (0.973 to 1.317)
		6	Inverse variance weighted	**0.0425**	1.131 (1.004 to 1.275)
		6	Simple mode	0.2714	1.124 (0.934 to 1.353)
		6	Weighted mode	0.1417	1.143 (0.984 to 1.328)
Isovalerylcarnitine (C5)	Idiopathic Pulmonary Fibrosis	4	MR Egger	0.4803	0.557 (0.147 to 2.114)
		4	Weighted median	0.0682	0.779 (0.596 to 1.019)
		4	Inverse variance weighted	**0.0240**	0.733 (0.559 to 0.960)
		4	Simple mode	0.5044	0.835 (0.523 to 1.333)
		4	Weighted mode	0.3803	0.832 (0.585 to 1.183)
Oleoyl-linoleoyl-glycerol (18:1/18:2) [2]	Idiopathic Pulmonary Fibrosis	7	MR Egger	0.5392	0.837 (0.492 to 1.422)
		7	Weighted median	0.1599	0.846 (0.670 to 1.068)
		7	Inverse variance weighted	**0.0341**	0.803 (0.656 to 0.984)
		7	Simple mode	0.5108	0.882 (0.621 to 1.254)
		7	Weighted mode	0.3403	0.861 (0.649 to 1.143)
Orotate	Idiopathic Pulmonary Fibrosis	4	MR Egger	0.2167	0.850 (0.711 to 1.016)
		4	Weighted median	**0.0221**	0.887 (0.800 to 0.983)
		4	Inverse variance weighted	**0.0170**	0.888 (0.805 to 0.979)
		4	Simple mode	0.1565	0.877 (0.764 to 1.006)
		4	Weighted mode	0.1189	0.884 (0.791 to 0.988)
Phosphate to oleoyl-linoleoyl-glycerol (18:1 to 18:2) [2]	Idiopathic Pulmonary Fibrosis	6	MR Egger	0.6656	1.113 (0.710 to 1.743)
		6	Weighted median	0.1282	1.211 (0.946 to 1.551)
		6	Inverse variance weighted	**0.0239**	1.248 (1.030 to 1.511)
		6	Simple mode	0.4647	1.148 (0.816 to 1.616)
		6	Weighted mode	0.3300	1.175 (0.876 to 1.576)
Retinol (Vitamin A) to oleoyl-linoleoyl-glycerol (18:1 to 18:2) [2]	Idiopathic Pulmonary Fibrosis	7	MR Egger	0.8908	1.036 (0.644 to 1.665)
		7	Weighted median	0.1657	1.182 (0.933 to 1.498)
		7	Inverse variance weighted	**0.0079**	1.330 (1.078 to 1.642)
		7	Simple mode	0.4236	1.154 (0.833 to 1.598)
		7	Weighted mode	0.3008	1.169 (0.892 to 1.532)
Stearoylcarnitine	Idiopathic Pulmonary Fibrosis	3	MR Egger	0.5957	0.668 (0.228 to 1.956)
		3	Weighted median	**0.0255**	0.702 (0.515 to 0.958)
		3	Inverse variance weighted	**0.0128**	0.712 (0.545 to 0.930)
		3	Simple mode	0.1642	0.653 (0.443 to 0.963)
		3	Weighted mode	0.1751	0.683 (0.476 to 0.981)
Tetradecadienedioate (C14:2-DC)	Idiopathic Pulmonary Fibrosis	3	MR Egger	0.6584	0.809 (0.402 to 1.628)
		3	Weighted median	**0.0021**	0.765 (0.645 to 0.907)
		3	Inverse variance weighted	**0.0007**	0.759 (0.647 to 0.891)
		3	Simple mode	0.1511	0.744 (0.576 to 0.960)
		3	Weighted mode	0.1031	0.769 (0.642 to 0.920)
Vanillic acid glycine	Idiopathic Pulmonary Fibrosis	4	MR Egger	0.9233	0.965 (0.512 to 1.821)
		4	Weighted median	0.1312	0.790 (0.582 to 1.073)
		4	Inverse variance weighted	**0.0434**	0.776 (0.606 to 0.993)
		4	Simple mode	0.1524	0.656 (0.425 to 1.011)
		4	Weighted mode	0.1826	0.667 (0.421 to 1.056)
X-11308	Idiopathic Pulmonary Fibrosis	3	MR Egger	0.6179	1.391 (0.541 to 3.580)
		3	Weighted median	**0.0200**	1.439 (1.059 to 1.954)
		3	Inverse variance weighted	**0.0273**	1.479 (1.045 to 2.095)
		3	Simple mode	0.2696	1.390 (0.907 to 2.129)
		3	Weighted mode	0.1530	1.438 (1.048 to 1.973)
X-11444	Idiopathic Pulmonary Fibrosis	7	MR Egger	0.4163	1.127 (0.865 to 1.467)
		7	Weighted median	0.0606	1.226 (0.991 to 1.517)
		7	Inverse variance weighted	**0.0079**	1.254 (1.061 to 1.481)
		7	Simple mode	0.1785	1.239 (0.940 to 1.632)
		7	Weighted mode	0.1622	1.207 (0.958 to 1.522)
X-11470	Idiopathic Pulmonary Fibrosis	7	MR Egger	0.2483	1.197 (0.914 to 1.568)
		7	Weighted median	**0.0486**	1.268 (1.001 to 1.605)
		7	Inverse variance weighted	**0.0051**	1.300 (1.082 to 1.563)
		7	Simple mode	0.2662	1.218 (0.888 to 1.671)
		7	Weighted mode	0.2209	1.228 (0.915 to 1.648)
X-16935	Idiopathic Pulmonary Fibrosis	5	MR Egger	0.0776	0.661 (0.486 to 0.899)
		5	Weighted median	**0.0115**	0.819 (0.701 to 0.956)
		5	Inverse variance weighted	**0.0055**	0.829 (0.726 to 0.946)
		5	Simple mode	0.3523	0.850 (0.629 to 1.150)
		5	Weighted mode	**0.0367**	0.766 (0.647 to 0.907)
X-21441	Idiopathic Pulmonary Fibrosis	7	MR Egger	0.3748	0.866 (0.648 to 1.157)
		7	Weighted median	0.0723	0.871 (0.750 to 1.013)
		7	Inverse variance weighted	**0.0313**	0.868 (0.763 to 0.987)
		7	Simple mode	0.1988	0.874 (0.727 to 1.049)
		7	Weighted mode	0.1362	0.872 (0.746 to 1.019)

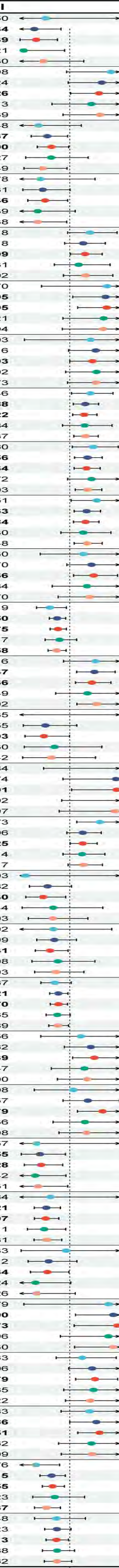

Fig. (S5).

Table S1. Heterogeneity of immune cells and IPF.

id.exposure	id.outcome	outcome	exposure	method	Q	Q_df	Q_pval
**qhgcCL**	HtaqDx	Idiopathic Pulmonary Fibrosis	CD39+ CD8br AC Treg	MR Egger	4.356894	4	0.359855
**qhgcCL**	HtaqDx	Idiopathic Pulmonary Fibrosis	CD39+ CD8br AC Treg	Inverse variance weighted	4.403612	5	0.492883
**HXoou4**	zYeUM0	Idiopathic Pulmonary Fibrosis	CD39+ CD8br Treg %CD8br Treg	MR Egger	5.221686	5	0.389429
**HXoou4**	zYeUM0	Idiopathic Pulmonary Fibrosis	CD39+ CD8br Treg %CD8br Treg	Inverse variance weighted	5.530898	6	0.47773
**6MP4aa**	8MMms9	Idiopathic Pulmonary Fibrosis	CD39+ CD8br Treg %T cell	MR Egger	6.126635	5	0.294091
**6MP4aa**	8MMms9	Idiopathic Pulmonary Fibrosis	CD39+ CD8br Treg %T cell	Inverse variance weighted	6.128118	6	0.408992
**lk7lnN**	jMejY8	Idiopathic Pulmonary Fibrosis	Activated Treg %CD4 Treg	MR Egger	0.016959	1	0.896387
**lk7lnN**	jMejY8	Idiopathic Pulmonary Fibrosis	Activated Treg %CD4 Treg	Inverse variance weighted	0.022758	2	0.988686
**6rNCcG**	Y7KZNq	Idiopathic Pulmonary Fibrosis	CD39+ CD4+ Treg %CD4+ Treg	MR Egger	4.016755	3	0.259661
**6rNCcG**	Y7KZNq	Idiopathic Pulmonary Fibrosis	CD39+ CD4+ Treg %CD4+ Treg	Inverse variance weighted	4.02706	4	0.402356
**zxZwRs**	fPNywa	Idiopathic Pulmonary Fibrosis	CD39+ CD4+ Treg %T cell	MR Egger	4.280312	4	0.369397
**zxZwRs**	fPNywa	Idiopathic Pulmonary Fibrosis	CD39+ CD4+ Treg %T cell	Inverse variance weighted	4.406483	5	0.492492
**OgiMjX**	FvBFlb	Idiopathic Pulmonary Fibrosis	Activated & resting Treg %CD4+ Treg	MR Egger	1.585264	1	0.208004
**OgiMjX**	FvBFlb	Idiopathic Pulmonary Fibrosis	Activated & resting Treg %CD4+ Treg	Inverse variance weighted	1.721362	2	0.422874
**upR3pv**	xXovqS	Idiopathic Pulmonary Fibrosis	HLA DR on CD33- HLA DR+ Myeloid cell	MR Egger	1.876558	1	0.170726
**upR3pv**	xXovqS	Idiopathic Pulmonary Fibrosis	HLA DR on CD33- HLA DR+ Myeloid cell	Inverse variance weighted	1.88593	2	0.389471
**e0PcXI**	t2361f	Idiopathic Pulmonary Fibrosis	CD39+ CD4+ AC Treg	MR Egger	4.684113	4	0.321271
**e0PcXI**	t2361f	Idiopathic Pulmonary Fibrosis	CD39+ CD4+ AC Treg	Inverse variance weighted	4.684922	5	0.455529

Table S2. Pleiotropy of immune cells and IPF.

id.exposure	id.outcome	outcome	exposure	egger_intercept	se	pval
**qhgcCL**	HtaqDx	Idiopathic Pulmonary Fibrosis	CD39+ CD8br AC Treg	0.009827	0.047448	0.846046
**HXoou4**	zYeUM0	Idiopathic Pulmonary Fibrosis	CD39+ CD8br Treg %CD8br Treg	0.02635	0.048426	0.609724
**6MP4aa**	8MMms9	Idiopathic Pulmonary Fibrosis	CD39+ CD8br Treg %T cell	-0.00177	0.050771	0.973595
**lk7lnN**	jMejY8	Idiopathic Pulmonary Fibrosis	Activated Treg %CD4 Treg	-0.00823	0.108097	0.951615
**6rNCcG**	Y7KZNq	Idiopathic Pulmonary Fibrosis	CD39+ CD4+ Treg %CD4+ Treg	-0.00554	0.063121	0.935621
**zxZwRs**	fPNywa	Idiopathic Pulmonary Fibrosis	CD39+ CD4+ Treg %T cell	-0.01899	0.055289	0.748603
**OgiMjX**	FvBFlb	Idiopathic Pulmonary Fibrosis	Activated & resting Treg %CD4+ Treg	-0.01184	0.040402	0.818547
**upR3pv**	xXovqS	Idiopathic Pulmonary Fibrosis	HLA DR on CD33- HLA DR+ Myeloid cell	-0.00902	0.127691	0.955085
**e0PcXI**	t2361f	Idiopathic Pulmonary Fibrosis	CD39+ CD4+ AC Treg	0.001443	0.054899	0.980293

Table S3. Heterogeneity of gut microbiota and IPF.

id.exposure	id.outcome	outcome	exposure	method	Q	Q_df	Q_pval
**ZTDaqH**	Nrah89	Idiopathic Pulmonary Fibrosis	Species CHKCI006 sp900018345	MR Egger	8.350358	11	0.681618
**ZTDaqH**	Nrah89	Idiopathic Pulmonary Fibrosis	Species CHKCI006 sp900018345	Inverse variance weighted	9.186022	12	0.686965
**kO8aPh**	ta5AFw	Idiopathic Pulmonary Fibrosis	Genus Escherichia	MR Egger	1.692064	7	0.974907
**kO8aPh**	ta5AFw	Idiopathic Pulmonary Fibrosis	Genus Escherichia	Inverse variance weighted	1.702812	8	0.988807
**x0g0Gc**	wcpPTI	Idiopathic Pulmonary Fibrosis	Species GCA-900199385 sp900320755	MR Egger	4.316111	6	0.633981
**x0g0Gc**	wcpPTI	Idiopathic Pulmonary Fibrosis	Species GCA-900199385 sp900320755	Inverse variance weighted	4.329944	7	0.741086
**2DR4hB**	7uNDlI	Idiopathic Pulmonary Fibrosis	Phylum Paceibacteria	MR Egger	2.534604	3	0.46907
**2DR4hB**	7uNDlI	Idiopathic Pulmonary Fibrosis	Phylum Paceibacteria	Inverse variance weighted	2.582569	4	0.629914

id.exposure	id.outcome	outcome	exposure	method	Q	Q_df	Q_pval
**CpIpYS**	jVaO6O	Idiopathic Pulmonary Fibrosis	Genus Phocea	MR Egger	3.074681	5	0.688473
**CpIpYS**	jVaO6O	Idiopathic Pulmonary Fibrosis	Genus Phocea	Inverse variance weighted	3.268893	6	0.77442
**7p3psy**	TvqBPU	Idiopathic Pulmonary Fibrosis	Species Rubneribacter sp002159915	MR Egger	0.859632	5	0.973075
**7p3psy**	TvqBPU	Idiopathic Pulmonary Fibrosis	Species Rubneribacter sp002159915	Inverse variance weighted	1.037072	6	0.984168
**VTuEu7**	ffXN7k	Idiopathic Pulmonary Fibrosis	Phylum SAR324	MR Egger	6.533702	8	0.587672
**VTuEu7**	ffXN7k	Idiopathic Pulmonary Fibrosis	Phylum SAR324	Inverse variance weighted	6.542208	9	0.684662
**OGhrgw**	fDON2n	Idiopathic Pulmonary Fibrosis	Species Desulfovibrio piger	MR Egger	7.286152	7	0.399706
**OGhrgw**	fDON2n	Idiopathic Pulmonary Fibrosis	Species Desulfovibrio piger	Inverse variance weighted	7.753002	8	0.457962
**ecNx9T**	s99ade	Idiopathic Pulmonary Fibrosis	Class Thermococci	Inverse variance weighted	5.27258	7	0.626737
**ecNx9T**	s99ade	Idiopathic Pulmonary Fibrosis	Class Thermococci	MR Egger	5.220811	6	0.51582

Table S4. Pleiotropy of gut microbiota and IPF.

id.exposure	id.outcome	outcome	exposure	egger_intercept	se	pval
**7p3psy**	TvqBPU	Idiopathic Pulmonary Fibrosis	Species Rubneribacter sp002159915	-0.01626	0.038598	0.691077
**kO8aPh**	ta5AFw	Idiopathic Pulmonary Fibrosis	Genus Escherichia	-0.00388	0.037462	0.920338
**2DR4hB**	7uNDlI	Idiopathic Pulmonary Fibrosis	Phylum Paceibacteria	0.019149	0.087435	0.840696
**VTuEu7**	ffXN7k	Idiopathic Pulmonary Fibrosis	Phylum SAR324	0.002583	0.028001	0.928784
**x0g0Gc**	wcpPTI	Idiopathic Pulmonary Fibrosis	Species GCA-900199385 sp900320755	0.003893	0.033097	0.910214
**ZTDaqH**	Nrah89	Idiopathic Pulmonary Fibrosis	Species CHKCI006 sp900018345	0.024599	0.02691	0.380241
**CpIpYS**	jVaO6O	Idiopathic Pulmonary Fibrosis	Genus Phocea	-0.01608	0.036479	0.677836
**ecNx9T**	s99ade	Idiopathic Pulmonary Fibrosis	Class Thermococci	0.018724	0.082294	0.827568
**OGhrgw**	fDON2n	Idiopathic Pulmonary Fibrosis	Species Desulfovibrio piger	-0.02236	0.033387	0.524509

Table S5. Heterogeneity of inflammatory proteins and IPF.

id.exposure	id.outcome	outcome	exposure	method	Q	Q_df	Q_pval
**WHFBiJ**	CMXU6o	Idiopathic Pulmonary Fibrosis	LIFR	MR Egger	1.412489	1	0.234644
**WHFBiJ**	CMXU6o	Idiopathic Pulmonary Fibrosis	LIFR	Inverse variance weighted	1.444387	2	0.485686
**i4PgkA**	ettZnB	Idiopathic Pulmonary Fibrosis	CCL25	MR Egger	2.636278	4	0.62041
**i4PgkA**	ettZnB	Idiopathic Pulmonary Fibrosis	CCL25	Inverse variance weighted	2.646286	5	0.754321
**bRU2AZ**	MPUOuK	Idiopathic Pulmonary Fibrosis	TNFSF12	MR Egger	4.423451	4	0.351721
**bRU2AZ**	MPUOuK	Idiopathic Pulmonary Fibrosis	TNFSF12	Inverse variance weighted	4.437993	5	0.488222

Table S6. Pleiotropy of inflammatory proteins and IPF.

id.exposure	id.outcome	outcome	exposure	egger_intercept	se	pval
**WHFBiJ**	CMXU6o	Idiopathic Pulmonary Fibrosis	LIFR	0.007133	0.047465	0.905041
**i4PgkA**	ettZnB	Idiopathic Pulmonary Fibrosis	CCL25	0.002717	0.027154	0.925126
**bRU2AZ**	MPUOuK	Idiopathic Pulmonary Fibrosis	TNFSF12	0.005013	0.043712	0.914229

Table S7. Heterogeneity of plasma lipidome and IPF.

id.exposure	id.outcome	outcome	exposure	method	Q	Q_df	Q_pval
**DBsDj2**	tLWIo8	Idiopathic Pulmonary Fibrosis	Sterol ester (27:1/18:3) levels	MR Egger	0.623931	3	0.890933
**DBsDj2**	tLWIo8	Idiopathic Pulmonary Fibrosis	Sterol ester (27:1/18:3) levels	Inverse variance weighted	2.053374	4	0.725942

Table S8. Pleiotropy of plasma lipidome and IPF.

id.exposure	id.outcome	outcome	exposure	egger_intercept	se	pval
**DBsDj2**	tLWIo8	Idiopathic Pulmonary Fibrosis	Sterol ester (27:1/18:3) levels	-0.04932	0.041249	0.317745

Table S9. Heterogeneity of plasma metabolites and IPF.

id.exposure	id.outcome	outcome	exposure	method	Q	Q_df	Q_pval
**uUZk0z**	gz6SKy	Idiopathic Pulmonary Fibrosis	X-11308 levels	MR Egger	3.800271	1	0.051244
**9A718z**	lvbjPc	Idiopathic Pulmonary Fibrosis	Isovalerylcarnitine (C5) levels	MR Egger	5.189458	2	0.074666
**9A718z**	lvbjPc	Idiopathic Pulmonary Fibrosis	Isovalerylcarnitine (C5) levels	Inverse variance weighted	5.637097	3	0.130665
**uUZk0z**	gz6SKy	Idiopathic Pulmonary Fibrosis	X-11308 levels	Inverse variance weighted	3.884268	2	0.143398
**lVzT4n**	G0Em9i	Idiopathic Pulmonary Fibrosis	Oleoyl-linoleoyl-glycerol (18:1/18:2) [2] levels	MR Egger	7.819857	5	0.166447
**2atMZy**	9eyvZi	Idiopathic Pulmonary Fibrosis	1-oleoylglycerol (18:1) levels	MR Egger	2.957758	2	0.227893
**Zt9CCe**	0RYSdI	Idiopathic Pulmonary Fibrosis	Retinol (Vitamin A) to oleoyl-linoleoyl-glycerol (18:1 to 18:2) [2] ratio	Inverse variance weighted	7.895249	6	0.245879
**lVzT4n**	G0Em9i	Idiopathic Pulmonary Fibrosis	Oleoyl-linoleoyl-glycerol (18:1/18:2) [2] levels	Inverse variance weighted	7.863145	6	0.248302
**lTgZXO**	dp9HzR	Idiopathic Pulmonary Fibrosis	Stearoylcarnitine levels	MR Egger	1.278952	1	0.258094
**Zt9CCe**	0RYSdI	Idiopathic Pulmonary Fibrosis	Retinol (Vitamin A) to oleoyl-linoleoyl-glycerol (18:1 to 18:2) [2] ratio	MR Egger	6.252396	5	0.282429
**HKRr0O**	zIKEPM	Idiopathic Pulmonary Fibrosis	Deoxycarnitine levels	MR Egger	3.724226	3	0.292824
**aZ1co6**	Xf3sDf	Idiopathic Pulmonary Fibrosis	X-16935 levels	Inverse variance weighted	4.260033	4	0.371957
**8o1eT3**	bjmyHC	Idiopathic Pulmonary Fibrosis	Alpha-ketoglutarate to proline ratio	MR Egger	1.949777	2	0.377234
**y8JVL0**	I4pdR5	Idiopathic Pulmonary Fibrosis	Decadienedioic acid (C10:2-D-C) levels	Inverse variance weighted	5.252295	5	0.385872
**8OqEas**	vJseFa	Idiopathic Pulmonary Fibrosis	Vanillic acid glycine levels	MR Egger	1.884406	2	0.389768
**2atMZy**	9eyvZi	Idiopathic Pulmonary Fibrosis	1-oleoylglycerol (18:1) levels	Inverse variance weighted	3.011327	3	0.389882
**urdfRe**	qbf82X	Idiopathic Pulmonary Fibrosis	Bilirubin degradation product, C17H18N2O4 (3) levels	MR Egger	0.726523	1	0.394013
**y8JVL0**	I4pdR5	Idiopathic Pulmonary Fibrosis	Decadienedioic acid (C10:2-D-C) levels	MR Egger	3.983865	4	0.408194
**HKRr0O**	zIKEPM	Idiopathic Pulmonary Fibrosis	Deoxycarnitine levels	Inverse variance weighted	3.793116	4	0.434728
**kXWrPg**	fUBYP7	Idiopathic Pulmonary Fibrosis	Cholesterol to oleoyl-li-noleoyl-glycerol (18:1 to 18:2) [2] ratio	MR Egger	6.549456	7	0.477245
**8OqEas**	vJseFa	Idiopathic Pulmonary Fibrosis	Vanillic acid glycine levels	Inverse variance weighted	2.42096	3	0.489745
**X4Xn74**	yRzwIo	Idiopathic Pulmonary Fibrosis	Isobutyrylcarnitine (c4) levels	Inverse variance weighted	4.307426	5	0.506055
**BzHDml**	6HEgJQ	Idiopathic Pulmonary Fibrosis	3-methoxytyrosine levels	MR Egger	3.253233	4	0.516376
**lTgZXO**	dp9HzR	Idiopathic Pulmonary Fibrosis	Stearoylcarnitine levels	Inverse variance weighted	1.298443	2	0.522452
**urdfRe**	qbf82X	Idiopathic Pulmonary Fibrosis	Bilirubin degradation product, C17H18N2O4 (3) levels	Inverse variance weighted	1.280484	2	0.527165
**b7kkrQ**	SDU15R	Idiopathic Pulmonary Fibrosis	Biliverdin levels	MR Egger	0.395949	1	0.529189
**ag3oyn**	cen30Q	Idiopathic Pulmonary Fibrosis	Bilirubin degradation product, C16H18N2O5 (3) levels	MR Egger	0.385091	1	0.534891
**b7kkrQ**	SDU15R	Idiopathic Pulmonary Fibrosis	Biliverdin levels	Inverse variance weighted	1.232865	2	0.539867
**ZzgvtX**	OK5NGX	Idiopathic Pulmonary Fibrosis	Tetradecadienedioate (C14:2-DC) levels	MR Egger	0.364942	1	0.545774
**kXWrPg**	fUBYP7	Idiopathic Pulmonary Fibrosis	Cholesterol to oleoyl-li-noleoyl-glycerol (18:1 to 18:2) [2] ratio	Inverse variance weighted	6.694479	8	0.569926

id.exposure	id.outcome	outcome	exposure	method	Q	Q_df	Q_pval
**8o1eT3**	bjmyHC	Idiopathic Pulmonary Fibrosis	Alpha-ketoglutarate to proline ratio	Inverse variance weighted	2.008836	3	0.570575
**BzHDml**	6HEgJQ	Idiopathic Pulmonary Fibrosis	3-methoxytyrosine levels	Inverse variance weighted	3.833638	5	0.573608
**AindNC**	8hQKp4	Idiopathic Pulmonary Fibrosis	Eicosenoylcarnitine (C20:1) levels	Inverse variance weighted	1.085573	2	0.581127
**ag3oyn**	cen30Q	Idiopathic Pulmonary Fibrosis	Bilirubin degradation product, C16H18N2O5 (3) levels	Inverse variance weighted	0.996792	2	0.607504
**aZ1co6**	Xf3sDf	Idiopathic Pulmonary Fibrosis	X-16935 levels	MR Egger	1.73849	3	0.628411
**L0VeLH**	XHti1v	Idiopathic Pulmonary Fibrosis	X-21441 levels	MR Egger	3.215209	5	0.666846
**k4yCnl**	o491g0	Idiopathic Pulmonary Fibrosis	X-11444 levels	Inverse variance weighted	3.34181	6	0.764883
**L0VeLH**	XHti1v	Idiopathic Pulmonary Fibrosis	X-21441 levels	Inverse variance weighted	3.215502	6	0.781353
**JAauCH**	ZDPPno	Idiopathic Pulmonary Fibrosis	X-11470 levels	MR Egger	2.359376	5	0.797508
**JAauCH**	ZDPPno	Idiopathic Pulmonary Fibrosis	X-11470 levels	Inverse variance weighted	3.037156	6	0.804169
**LYslN1**	vtGEcg	Idiopathic Pulmonary Fibrosis	Phosphate to oleoyl-li-noleoyl-glycerol (18:1 to 18:2) [2] ratio	MR Egger	1.623253	4	0.804607
**wMoiXf**	f7Z2T2	Idiopathic Pulmonary Fibrosis	Argininate levels	MR Egger	0.985816	3	0.804684
**k4yCnl**	o491g0	Idiopathic Pulmonary Fibrosis	X-11444 levels	MR Egger	2.292584	5	0.807355
**ZzgvtX**	OK5NGX	Idiopathic Pulmonary Fibrosis	Tetradecadienedioate (C14:2-DC) levels	Inverse variance weighted	0.398084	2	0.819515
**e6qLge**	Ty61Ko	Idiopathic Pulmonary Fibrosis	11beta-hydroxyandrosterone glucuronide levels	MR Egger	0.842373	3	0.839307
**LYslN1**	vtGEcg	Idiopathic Pulmonary Fibrosis	Phosphate to oleoyl-li-noleoyl-glycerol (18:1 to 18:2) [2] ratio	Inverse variance weighted	1.928278	5	0.858978
**e6qLge**	Ty61Ko	Idiopathic Pulmonary Fibrosis	11beta-hydroxyandrosterone glucuronide levels	Inverse variance weighted	1.216645	4	0.875351
**SxBPTb**	j4YSyk	Idiopathic Pulmonary Fibrosis	Gamma-glutamyl-2-aminobu-tyrate levels	Inverse variance weighted	0.24652	2	0.884034
**X4Xn74**	yRzwIo	Idiopathic Pulmonary Fibrosis	Isobutyrylcarnitine (c4) levels	MR Egger	1.151243	4	0.886059
**KjVqnJ**	7ybbJt	Idiopathic Pulmonary Fibrosis	Orotate levels	MR Egger	0.232059	2	0.890449
**KjVqnJ**	7ybbJt	Idiopathic Pulmonary Fibrosis	Orotate levels	Inverse variance weighted	0.554016	3	0.906874
**wMoiXf**	f7Z2T2	Idiopathic Pulmonary Fibrosis	Argininate levels	Inverse variance weighted	0.987453	4	0.911693
**SxBPTb**	j4YSyk	Idiopathic Pulmonary Fibrosis	Gamma-glutamyl-2-aminobu-tyrate levels	MR Egger	0.003874	1	0.950372
**jGNBmz**	yy4dfe	Idiopathic Pulmonary Fibrosis	Arachidoylcarnitine (C20) lev-els	MR Egger	0.088477	2	0.956726
**jhNGaZ**	j1R07e	Idiopathic Pulmonary Fibrosis	Androsterone glucuronide to etiocholanolone glucuronide ratio	MR Egger	1.773923	7	0.971288
**jhNGaZ**	j1R07e	Idiopathic Pulmonary Fibrosis	Androsterone glucuronide to etiocholanolone glucuronide ratio	Inverse variance weighted	2.040108	8	0.979758
**AindNC**	8hQKp4	Idiopathic Pulmonary Fibrosis	Eicosenoylcarnitine (C20:1) levels	MR Egger	0.000408	1	0.983876
**jGNBmz**	yy4dfe	Idiopathic Pulmonary Fibrosis	Arachidoylcarnitine (C20) lev-els	Inverse variance weighted	0.088718	3	0.993156

Table S10. Pleiotropy of plasma metabolites and IPF.

id.exposure	id.outcome	outcome	exposure	egger_intercept	se	pval
**ZzgvtX**	OK5NGX	Idiopathic Pulmonary Fibrosis	Tetradecadienedioate (C14:2-DC) levels	-0.01811	0.099479	0.885359
**2atMZy**	9eyvZi	Idiopathic Pulmonary Fibrosis	1-oleoylglycerol (18:1) levels	-0.02266	0.119084	0.866624
**JAauCH**	ZDPPno	Idiopathic Pulmonary Fibrosis	X-11470 levels	0.027871	0.033853	0.447825

id.exposure	id.outcome	outcome	exposure	egger_intercept	se	pval
**aZ1co6**	Xf3sDf	Idiopathic Pulmonary Fibrosis	X-16935 levels	0.052495	0.033058	0.210505
**urdfRe**	qbf82X	Idiopathic Pulmonary Fibrosis	Bilirubin degradation product, C17H18N2O4 (3) levels	-0.0216	0.02902	0.592669
**HKRr0O**	zIKEPM	Idiopathic Pulmonary Fibrosis	Deoxycarnitine levels	-0.00716	0.030391	0.828932
**y8JVL0**	I4pdR5	Idiopathic Pulmonary Fibrosis	Decadienedioic acid (C10:2-D-C) levels	0.037172	0.033006	0.323057
**Zt9CCe**	0RYSdI	Idiopathic Pulmonary Fibrosis	Retinol (Vitamin A) to oleoyl-linoleoyl-glycerol (18:1 to 18:2) [2] ratio	0.037913	0.033077	0.303588
**k4yCnl**	o491g0	Idiopathic Pulmonary Fibrosis	X-11444 levels	0.037591	0.036698	0.352663
**b7kkrQ**	SDU15R	Idiopathic Pulmonary Fibrosis	Biliverdin levels	-0.03511	0.038378	0.528297
**jGNBmz**	yy4dfe	Idiopathic Pulmonary Fibrosis	Arachidoylcarnitine (C20) lev-els	-0.00072	0.046267	0.989021
**ag3oyn**	cen30Q	Idiopathic Pulmonary Fibrosis	Bilirubin degradation product, C16H18N2O5 (3) levels	-0.02087	0.026678	0.577451
**lTgZXO**	dp9HzR	Idiopathic Pulmonary Fibrosis	Stearoylcarnitine levels	0.00975	0.078975	0.921804
**kXWrPg**	fUBYP7	Idiopathic Pulmonary Fibrosis	Cholesterol to oleoyl-li-noleoyl-glycerol (18:1 to 18:2) [2] ratio	0.011682	0.030675	0.714631
**KjVqnJ**	7ybbJt	Idiopathic Pulmonary Fibrosis	Orotate levels	0.018674	0.03291	0.627632
**LYslN1**	vtGEcg	Idiopathic Pulmonary Fibrosis	Phosphate to oleoyl-li-noleoyl-glycerol (18:1 to 18:2) [2] ratio	0.018422	0.033356	0.610156
**9A718z**	lvbjPc	Idiopathic Pulmonary Fibrosis	Isovalerylcarnitine (C5) levels	0.042604	0.102574	0.718203
**uUZk0z**	gz6SKy	Idiopathic Pulmonary Fibrosis	X-11308 levels	0.010821	0.072784	0.906041
**L0VeLH**	XHti1v	Idiopathic Pulmonary Fibrosis	X-21441 levels	0.000629	0.036702	0.986993
**lVzT4n**	G0Em9i	Idiopathic Pulmonary Fibrosis	Oleoyl-linoleoyl-glycerol (18:1/18:2) [2] levels	-0.00644	0.038723	0.874386
**8o1eT3**	bjmyHC	Idiopathic Pulmonary Fibrosis	Alpha-ketoglutarate to proline ratio	0.012375	0.050921	0.830641
**SxBPTb**	j4YSyk	Idiopathic Pulmonary Fibrosis	Gamma-glutamyl-2-aminobu-tyrate levels	-0.03906	0.079299	0.708617
**jhNGaZ**	j1R07e	Idiopathic Pulmonary Fibrosis	Androsterone glucuronide to etiocholanolone glucuronide ratio	-0.01298	0.025162	0.621794
**BzHDml**	6HEgJQ	Idiopathic Pulmonary Fibrosis	3-methoxytyrosine levels	0.026485	0.034764	0.488598
**X4Xn74**	yRzwIo	Idiopathic Pulmonary Fibrosis	Isobutyrylcarnitine (c4) levels	-0.04723	0.026585	0.150285
**e6qLge**	Ty61Ko	Idiopathic Pulmonary Fibrosis	11beta-hydroxyandrosterone glucuronide levels	-0.02274	0.037172	0.583928
**8OqEas**	vJseFa	Idiopathic Pulmonary Fibrosis	Vanillic acid glycine levels	-0.03596	0.049096	0.540077
**wMoiXf**	f7Z2T2	Idiopathic Pulmonary Fibrosis	Argininate levels	0.003058	0.075569	0.970265
**AindNC**	8hQKp4	Idiopathic Pulmonary Fibrosis	Eicosenoylcarnitine (C20:1) levels	0.097614	0.093706	0.486996

Table S11. Heterogeneity of immune cells and potential mediators.

id.exposure	id.outcome	outcome	exposure	method	Q	Q_df	Q_pval
**ebi-a-GCST90001487**	koGehr	Isobutyrylcarnitine (c4)	Activated Treg %CD4 Treg	MR Egger	0.071309	1	0.78944
**ebi-a-GCST90001487**	koGehr	Isobutyrylcarnitine (c4)	Activated Treg %CD4 Treg	Inverse variance weighted	0.074505	2	0.963433
**ebi-a-GCST90002112**	MqZZ0d	Arachidoylcarnitine (C20)	HLA DR on CD33- HLA DR+	MR Egger	0.226766	1	0.633932
**ebi-a-GCST90002112**	MqZZ0d	Arachidoylcarnitine (C20)	HLA DR on CD33- HLA DR+	Inverse variance weighted	0.611141	2	0.736703
**ebi-a-GCST90001671**	csAsCf	X-16935	CD39+ CD8br %CD8br	MR Egger	3.094287	4	0.542173

id.exposure	id.outcome	outcome	exposure	method	Q	Q_df	Q_pval
**ebi-a-GCST90001671**	csAsCf	X-16935	CD39+ CD8br %CD8br	Inverse variance weighted	3.999204	5	0.549531
**ebi-a-GCST90002112**	flU1zs	Decadienedioic acid (C10:2-DC)	HLA DR on CD33- HLA DR+	MR Egger	1.032642	1	0.309539
**ebi-a-GCST90002112**	flU1zs	Decadienedioic acid (C10:2-DC)	HLA DR on CD33- HLA DR+	Inverse variance weighted	1.396401	2	0.49748
**ebi-a-GCST90001487**	1s4QXZ	CCL25	Activated Treg %CD4 Treg	MR Egger	0.41268	1	0.520612
**ebi-a-GCST90001487**	1s4QXZ	CCL25	Activated Treg %CD4 Treg	Inverse variance weighted	2.222419	2	0.329161
**ebi-a-GCST90002112**	ARfTHp	LIFR	HLA DR on CD33- HLA DR+	MR Egger	0.17088	2	0.918108
**ebi-a-GCST90002112**	ARfTHp	LIFR	HLA DR on CD33- HLA DR+	Inverse variance weighted	4.732668	3	0.192452
**ebi-a-GCST90001660**	ARfTHp	LIFR	CD39+ CD4+ AC	MR Egger	5.226045	4	0.26488
**ebi-a-GCST90001660**	ARfTHp	LIFR	CD39+ CD4+ AC	Inverse variance weighted	5.954814	5	0.310641
**ebi-a-GCST90001487**	iPWmgF	TNFSF12	Activated Treg %CD4 Treg	MR Egger	0.09024	1	0.763872
**ebi-a-GCST90001487**	iPWmgF	TNFSF12	Activated Treg %CD4 Treg	Inverse variance weighted	0.266154	2	0.875398
**ebi-a-GCST90001487**	bBjfwB	Phylum SAR324	Activated Treg %CD4 Treg	MR Egger	0.013825	1	0.9064
**ebi-a-GCST90001487**	bBjfwB	Phylum SAR324	Activated Treg %CD4 Treg	Inverse variance weighted	0.017389	2	0.991343
**ebi-a-GCST90001658**	UOmcnl	Species CHKCI006 sp900018345	CD39+ CD4+ %T cell	MR Egger	5.052269	3	0.16801
**ebi-a-GCST90001658**	UOmcnl	Species CHKCI006 sp900018345	CD39+ CD4+ %T cell	Inverse variance weighted	5.178231	4	0.269494

Table S12. Pleiotropy of immune cells and potential mediators.

id.exposure	id.outcome	outcome	exposure	egger_intercept	se	pval
**ebi-a-GCST90001487**	koGehr	Isobutyrylcarnitine (c4)	Activated Treg %CD4 Treg	0.003621	0.064057	0.964049
**ebi-a-GCST90002112**	MqZZ0d	Arachidoylcarnitine (C20)	HLA DR on CD33- HLA DR+	0.032144	0.051846	0.646688
**ebi-a-GCST90001671**	csAsCf	X-16935	CD39+ CD8br %CD8br	0.024439	0.025691	0.395334
**ebi-a-GCST90002112**	flU1zs	Decadienedioic acid (C10:2-DC)	HLA DR on CD33- HLA DR+	0.029917	0.050406	0.659002
**ebi-a-GCST90001487**	1s4QXZ	CCL25	Activated Treg %CD4 Treg	0.077265	0.057435	0.406946
**ebi-a-GCST90002112**	ARfTHp	LIFR	HLA DR on CD33- HLA DR+	-0.08996	0.042121	0.16621
**ebi-a-GCST90001660**	ARfTHp	LIFR	CD39+ CD4+ AC	0.019001	0.025441	0.496657
**ebi-a-GCST90001487**	iPWmgF	TNFSF12	Activated Treg %CD4 Treg	-0.0243	0.057947	0.747176
**ebi-a-GCST90001487**	bBjfwB	Phylum SAR324	Activated Treg %CD4 Treg	-0.00083	0.013902	0.962041
**ebi-a-GCST90001658**	UOmcnl	Species CHKCI006 sp900018345	CD39+ CD4+ %T cell	0.006562	0.023992	0.802227

Table S13. Genes significantly associated with immune cells and IPF.

id	chr	start	end	strand	gene_ids	gene_ names	3_prime _utr_variant	nmd_transcript _variant	Intron _variant	non_coding_ transcript_ exon_variant	non_coding _transcript_variant
**rs138915779**	1	1.9E+08	1.9E+08	+	ENSG00000162670	BRINP3	0	0	1	0	0
**rs6751481**	2	38670668	38670668	+	ENSG00000143891, ENSG00000232518	GALM	0	2	10	0	4
**rs408686**	2	38950179	38950179	+	ENSG00000214694	ARHGEF33	0	0	6	0	0
**rs145557266**	10	94090298	94090298	+	ENSG00000138193, ENSG00000232913	PLCE1	0	4	15	0	3
**rs4918960**	10	95712813	95712813	+	ENSG00000138185	ENTPD1	0	0	3	0	0
**rs12778797**	10	95238931	95238931	+	ENSG00000107438	PDLIM1	0	0	6	0	4

id	chr	start	end	strand	gene_ids	gene_ names	3_prime _utr_variant	nmd_transcript _variant	Intron _variant	non_coding_ transcript_ exon_variant	non_coding _transcript_variant
**rs10882701**	10	95981314	95981314	+	ENSG00000188649, ENSG00000226688	CC2D2B	0	2	14	0	10
**rs114844216**	10	96399195	96399195	+	ENSG00000095587	TLL2	0	0	2	0	1
**rs11818051**	10	96212538	96212538	+	ENSG00000095585	BLNK	0	10	19	0	0
**rs117648214**	10	97056677	97056677	+	ENSG00000187122	SLIT1	0	0	3	0	0
**rs114068468**	6	33086686	33086686	+	ENSG00000223865	HLA-DPB1	5	1	0	1	0
**rs9268430**	6	32377652	32377652	+	ENSG00000225914	TSBP1-AS1	0	0	13	0	13
